# Production of abscisic acid in the oleaginous yeast *Yarrowia lipolytica*

**DOI:** 10.1093/femsyr/foac015

**Published:** 2022-03-11

**Authors:** Jonathan Asmund Arnesen, Irene Hjorth Jacobsen, Jane Dannow Dyekjær, Daniela Rago, Mette Kristensen, Andreas Koedfoed Klitgaard, Milica Randelovic, José Luis Martinez, Irina Borodina

**Affiliations:** The Novo Nordisk Foundation Center for Biosustainability, Technical University of Denmark, Kemitorvet 220, 2800 Kgs Lyngby, Denmark; Department of Biotechnology and Biomedicine, Technical University of Denmark, Søltofts Plads 223, 2800 Kgs Lyngby, Denmark; The Novo Nordisk Foundation Center for Biosustainability, Technical University of Denmark, Kemitorvet 220, 2800 Kgs Lyngby, Denmark; The Novo Nordisk Foundation Center for Biosustainability, Technical University of Denmark, Kemitorvet 220, 2800 Kgs Lyngby, Denmark; The Novo Nordisk Foundation Center for Biosustainability, Technical University of Denmark, Kemitorvet 220, 2800 Kgs Lyngby, Denmark; The Novo Nordisk Foundation Center for Biosustainability, Technical University of Denmark, Kemitorvet 220, 2800 Kgs Lyngby, Denmark; The Novo Nordisk Foundation Center for Biosustainability, Technical University of Denmark, Kemitorvet 220, 2800 Kgs Lyngby, Denmark; Department of Biotechnology and Biomedicine, Technical University of Denmark, Søltofts Plads 223, 2800 Kgs Lyngby, Denmark; The Novo Nordisk Foundation Center for Biosustainability, Technical University of Denmark, Kemitorvet 220, 2800 Kgs Lyngby, Denmark

**Keywords:** abscisic acid, *Yarrowia lipolytica*, terpenoids, yeast, isoprenoids

## Abstract

Abscisic acid (ABA) is a phytohormone with applications in agriculture and human health. ABA can be produced by *Botrytis cinerea*, a plant pathogenic filamentous fungus. However, the cultivation process is lengthy and strain improvement by genetic engineering is difficult. Therefore, we engineered the oleaginous yeast *Yarrowia lipolytica* as an alternative host for ABA production. First, we expressed five *B. cinerea* genes involved in ABA biosynthesis (*BcABA1*,*BcABA2*,*BcABA3*,*BcABA4* and *BcCPR1*) in a *Y. lipolytica* chassis with optimized mevalonate flux. The strain produced 59.2 mg/L of ABA in small-scale cultivation. Next, we expressed an additional copy of each gene in the strain, but only expression of additional copy of *BcABA1* gene increased the ABA titer to 168.5 mg/L. We then integrated additional copies of the mevalonate pathway and ABA biosynthesis encoding genes, and we expressed plant ABA transporters resulting in an improved strain producing 263.5 mg/L and 9.1 mg/g dry cell weight (DCW) ABA. Bioreactor cultivation resulted in a specific yield of 12.8 mg/g DCW ABA; however, surprisingly, the biomass level obtained in bioreactors was only 10.5 g DCW/L, with a lower ABA titer of 133.6 mg/L. While further optimization is needed, this study confirms *Y. lipolytica* as a potential alternative host for the ABA production.

## Teaser

Engineering of the oleaginous yeast *Yarrowia lipolytica* for high-level abscisic acid production.

## Introduction

Abscisic acid (ABA) is a sesquiterpenoid phytohormone involved in plant developmental processes, such as seed dormancy, cell elongation and flower induction. It is also produced in response to abiotic stressors, e.g. drought or salt stress, and biotic stressors such as pathogen infection (Finkelstein 2013, Alazem and Lin 2017, Ma *et al*. 2018). Currently, ABA is used in agriculture to enhance the color of red table grapes and produce hybrid seeds (Shi *et al*. 2017). Furthermore, it has been demonstrated that ABA applications can enhance salt tolerance in citrus, drought tolerance in chickpeas and cold/drought tolerance in wheat (Gómez-Cadenas *et al*. 2002, Nayyar *et al*. 2005, Bano *et al*. 2012, Li *et al*. 2014). ABA also has potential as a nutraceutical and pharmaceutical agent. ABA is naturally present in animals such as sponges, rats, pigs and humans (Le Page-Degivry *et al*. 1986, Zocchi *et al*. 2001, 2017, Bruzzone *et al*. 2008). Dietary supplementation with ABA-rich fruit extracts decreased insulin and blood sugar levels (Magnone *et al*. 2015). Furthermore, ABA supplementation in mice improved host response to malaria infection (Glennon *et al*. 2016, 2018). Higher plasma levels of ABA correlate with a lower risk of malaria symptoms in children infected with *Plasmodium falciparum* (Glennon *et al*. 2018).

Fungal production of ABA was first reported in cell cultures of the plant pathogenic fungus *Cercospora rosicola* (Assante *et al*. 1977). Later, ABA production was discovered in other fungi species, such as *Botrytis cinerea*, *Fusarium oxysporum*, *Ceratocystis fimbriata*, *Ceratocystis coerulescens*, *Rhizoctonia solani* and *Magnaporthe oryzae* (Marumo *et al*. 1982, Dörffling *et al*. 1984, Spence *et al*. 2015). Fungi produce ABA during the infection process to alter the immune response of the host plant (Lievens *et al*. 2017). Some bacteria can also produce ABA, including certain strains from the *Pseudomonas* and *Bacillus* genera (Forchetti *et al*. 2007, Salomon *et al*. 2014). Cultivation of *B. cinerea* has been used for ABA production, with up to 2 g/L ABA titers reported (Gong *et al*. 2014, Ding *et al*. 2016, Shi *et al*. 2017). The high-producing *B. cinerea* strains were obtained by mutagenesis. There are only a few reports on rationally genetically engineered *B. cinerea* strains due to the lack of convenient genetic tools (Ding *et al*. 2015, Leisen *et al*. 2020).

**Figure 1.**
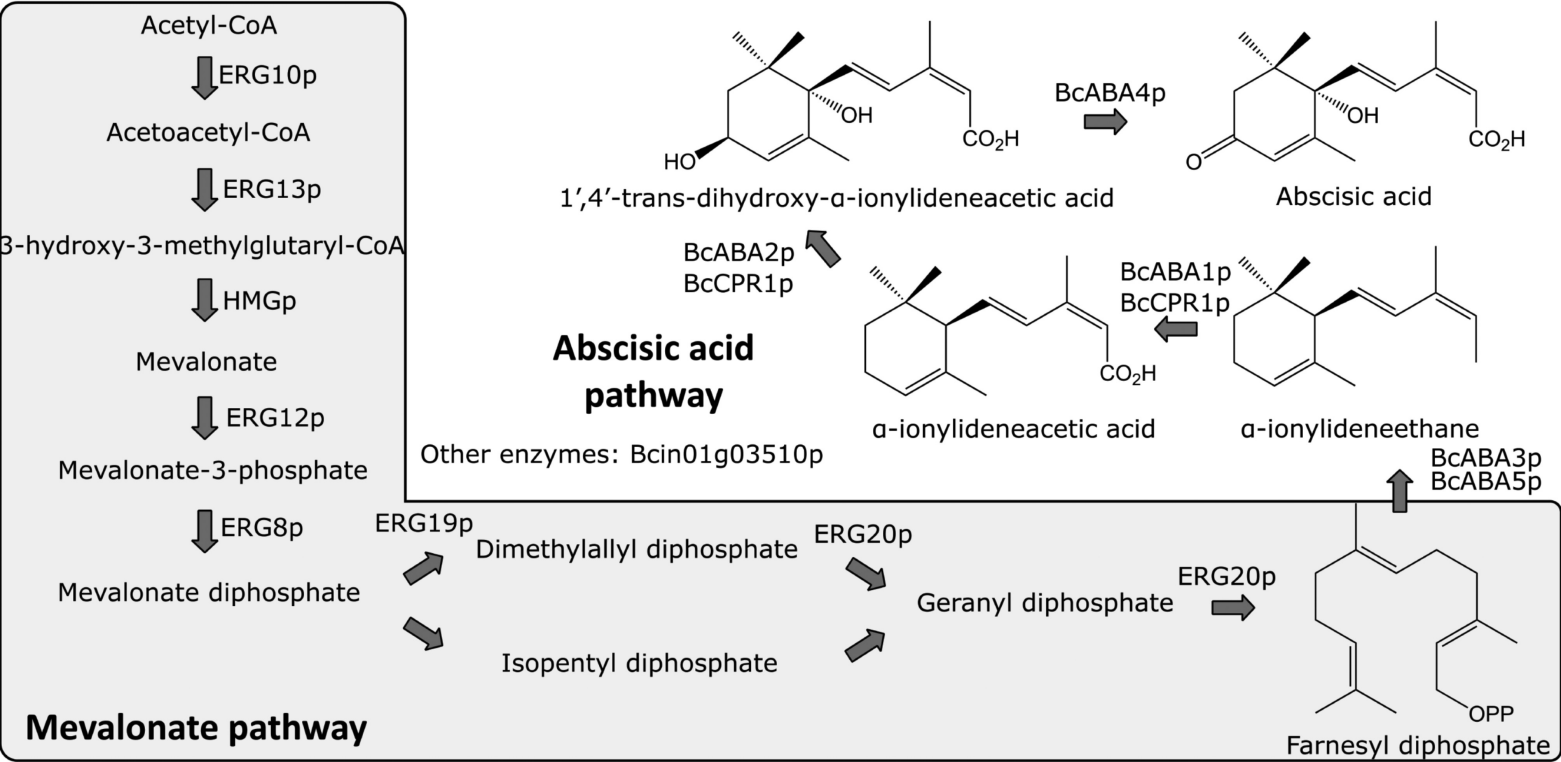
The native mevalonate (MVA) and heterologous ABA pathways. ERG10p, acetyl-CoA acetyltransferase; ERG13p, 3-hydroxy-3-methylglutaryl synthase; HMGp, 3-hydroxy-3-methylglutaryl reductase; ERG12p, mevalonate kinase; ERG8p, phosphomevalonate kinase; ERG19p, mevalonate diphosphate decarboxylase; ERG20p, farnesyl diphosphate synthase; BcABA3p, *B. cinerea* α-ionylideneethane synthases; BcABA5p, *B. cinerea* terpene synthase putatively involved in ABA biosynthesis; BcABA1p, *B. cinerea* cytochrome P450; BcABA2p, *B. cinerea* cytochrome P450; BcABA4p, *B. cinerea* dehydrogenase; BcCPR1p, *B. cinerea* cytochrome P450 reductase; and Bcin01g03510p, cytochrome P450 potentially involved in ABA biosynthesis.

The ABA biosynthetic pathway in *B. cinerea* was recently elucidated, representing the first comprehensive characterization of a fungal ABA biosynthetic pathway (Takino *et al*. 2019). In fungi, ABA is produced from the phosphorylated C_15_-terpene precursor farnesyl diphosphate (FPP), as opposed to plant and human cells where ABA is biosynthesized from carotenoid precursors derived from geranylgeranyl diphosphate (Bennett *et al*. 1984, Inomata *et al*. 2004, Zocchi *et al*. 2017). In *B. cinerea*, FPP is cyclized into the ABA precursor α-ionylideneethane by the α-ionylideneethane synthase (BcABA3p) (Fig. 1) (Takino *et al*. 2018). BcAba3p does not carry any terpene synthase-like motifs and may represent a new type of sesquiterpene cyclase. Subsequently, α-ionylideneethane is carboxylated by a cytochrome P450 (BcABA1p) into α-ionylideneacetic acid, which is then oxygenated by another cytochrome P450 (BcABA2p) forming 1′,4′-*trans*-dihydroxy-α-ionylideneacetic acid and lastly dehydrogenated by a dehydrogenase (BcABA4p) into ABA (Inomata *et al*. 2004, Takino *et al*. 2019). Interestingly, the four genes *BcABA1–4* form a gene cluster on chromosome 8 in the *B. cinerea* genome (Siewers *et al*. 2006, Van Kan *et al*. 2017). It was shown that the refactored expression of the genes *BcABA1–4* in *Aspergillus oryzae* resulted in the production of 8 mg/L ABA (Takino *et al*. 2018). Likewise, expression of single copies of *BcABA3–4*, the *B. cinerea* cytochrome P450 reductase *BcCPR1* and double copies of *BcABA1–2* in a pre-engineered *Saccharomyces cerevisiae* strain resulted in 11 mg/L ABA (Otto *et al*. 2019). The pre-engineered strain featured deletions of the genes encoding diacylglycerol pyrophosphatase, lipid phosphate phosphatase, downregulation of the squalene synthase gene, and overexpression of the FPP synthase (*ERG20*) and truncated 3-hydroxy-3-methylglutaryl-CoA reductase genes (López *et al*. 2015). These results suggest that the current heterologous hosts are suboptimal for ABA production. Therefore, we aimed to establish production in a host better suited for terpenoid production and amenable for large-scale cultivation. We chose to work with *Yarrowia lipolytica*, which has a high cytosolic acetyl-CoA pool available for terpenoid synthesis, features an excellent safety profile and is relatively easy to engineer (Dujon *et al*. 2004, Christen and Sauer 2011, Groenewald *et al*. 2014, Holkenbrink *et al*. 2018). Moreover, this yeast has been previously successfully engineered for the production of a range of terpenoids, such as α-farnesene, lycopene, d-limonene, betulinic acid, valencene, astaxanthin, insect pheromones and gibberellins (Matthäus *et al*. 2014, Cao *et al*. 2016, Yang *et al*. 2016, Gao *et al*. 2017, Kildegaard *et al*. 2017, 2021, Sun *et al*. 2019, Tramontin *et al*. 2019, Arnesen *et al*. 2020, Holkenbrink *et al*. 2020, Petkevicius *et al*. 2021). Indeed, several genetically modified *Y. lipolytica* strains have received GRAS (generally recognized as safe) status, including strains for the production of omega-3 fatty acids and steviol glycosides (Turck *et al*. 2019). Therefore, we sought to utilize *Y. lipolytica* to produce ABA and improve the production through genetic engineering and cultivation optimization.

## Methods

### Strains and media

A previously reported *Y. lipolytica* strain ST9149 engineered for improved MVA pathway flux toward FPP (Table S1, Supporting Information) was used to construct the ABA production strains (Arnesen *et al*. 2020). This strain was based on the W29-derived strain ST6512 (*MATa ku70∆::PrTEF1-Cas9-TTef12::PrGPD-DsdA-TLip2*) expressing CRISPR-based (clustered regularly interspaced short palindromic repeats) associated protein 9 (Cas9p) for DNA integration (Holkenbrink *et al*. 2018, Marella *et al*. 2020). ST6512 was derived from *Y. lipolytica* strain W29 (Y-63746), a kind gift from the ARS Culture Collection, National Center for Agricultural Utilization Research (NCAUR), USA. The DH5α *Escherichia coli* strain was used for plasmid construction. Lysogeny broth media with 100 mg/L ampicillin was used to cultivate *E. coli* cells at 300 rpm shaking and 37°C. The *Y. lipolytica *cells were cultivated at 30°C on media containing 10 g/L yeast extract, 20 g/L peptone and 20 g/L glucose (YPD) with 20 g/L agar added for solid media. Hygromycin (400 mg/L) or nourseothricin (250 mg/L) was added to the media for yeast cell selection. All chemicals were purchased from Sigma-Aldrich/Merck (Germany), unless otherwise noted. Nourseothricin was obtained from Jena BioScience GmbH (Germany). Rich liquid media with different nutrient formulations were prepared by varying the concentrations of yeast extract (Y, g/L), peptone (P, g/L) and glucose (D, g/L). The following media formulations were prepared: Y10P20D20 (standard YPD media), Y20P40D20, Y2.5P5D80, Y5P10D80, Y10P20D80 and Y20P40D80. The mineral medium was prepared with 0.5 g/L MgSO_4_⋅7H_2_O, 3 g/L KH_2_PO_4_, 5 g/L (NH_4_)_2_SO_4_, 0.1% (v/v) vitamin solution, 0.2% (v/v) trace metal solution and 80 g/L glucose as described in Sáez-Sáez *et al*. (2020).

### Plasmids

The plasmids, biobricks and primers used in this study are listed in Tables S2, S3 and S4 (Supporting Information), respectively. Phusion U polymerase (Thermo Scientific) was used to amplify the biobricks with polymerase chain reactions (PCR), which were assembled into EasyCloneYALI plasmids by Uracil-Specific Excision Reagent (USER) cloning (Holkenbrink *et al*. 2018). The USER reactions were transformed into *E. coli* and correct assembly was verified by sequencing. The genes encoding *BcABA1* (NCBI reference: XP_024550391.1), *BcABA2* (NCBI reference: XP_024550390.1), *BcABA3* (NCBI reference: XP_024550392.1), *BcABA4* (NCBI reference: XP_001553969.2), *BcCPR1* (NCBI reference: XP_001558194.1), a putative *B. cinerea* α-ionylideneethane synthase *BcABA5* (amino sequence as from strain ATCC58025 described in Izquierdo-Bueno *et al*. 2018), and the *Arabidopsis thaliana* ABA transporters *AtDTX50* and *AtABCG25* (UniProt references: Q9FJ87 and Q84TH5, respectively) were ordered as GeneArt Strings DNA fragments from Thermo Fischer Scientific. The DNA sequences were codon-optimized for *Y. lipolytica* using GeneArt standard algorithm. The DNA sequences are provided in the Supporting Information.

### Strain construction

Yeast strains are listed in Table S3 (Supporting Information). The integration vectors were *Not*I-digested before lithium acetate transformation as described earlier (Holkenbrink *et al*. 2018). The genomic integration of the plasmids was confirmed by colony PCR with primers complementary to the genomic region and plasmid (Holkenbrink *et al*. 2018).

### Cultivation

For precultures, 2.5 mL of Y10P20D20 in 24-well plates with an air-penetrable lid (EnzyScreen, NL) was inoculated with single yeast clones and grown for 16–24 h at 30°C and 300 rpm agitation. The optical density at 600 nm (OD600) was measured with a VWR NanoPhotometer 7122. Culture volume corresponding to 0.1 OD600 units was inoculated into 2.5 mL of Y10P20D80 or other media formulations in a 24-deep-well plate, which was incubated for 72 h at 30°C with 300 rpm agitation. All cultivations were performed in biological triplicates. Dry cell weight (DCW) was measured at the end of cultivation by transferring 0.5 or 1 mL of culture broth to a pre-weighed 2-mL microtube (Sarstedt). The samples were centrifuged and the supernatant was removed. The cell pellets were then dried at 60°C for 72 h.

### Sample preparation for ABA analysis

For quantification of intracellular ABA concentration, 1 mL of culture broth was transferred to a 2-mL microtube (Sarstedt). The samples were centrifuged at 16 000 g for 5 min, and the supernatant was removed. The cell pellets were resuspended in water, centrifuged at 16 000 g for 5 min, and the supernatant was removed. This washing step was repeated. One milliliter of acetonitrile and 500 µL of 0.212–0.3 mm acid-washed glass beads were added to each tube. Thereafter, the cells were disrupted with a Precellys^®^ 24 homogenizer (Bertin Corp.) using four cycles at 5500 rpm for 10 s each. The samples were then shaken for 10 min in a DVX-2500 Multi-Tube Vortexer (VWR, USA) at room temperature. Lastly, the samples were centrifuged at 16 000 g for 5 min, and the supernatant was collected for analysis.

For quantification of extracellular ABA concentration, 1 mL of culture broth was centrifuged at 16 000 g for 5 min and the supernatant was collected. Some samples were diluted with water before analysis.

### Analytical methods

All ABA concentrations were measured on a Dionex 3000 high performance liquid chromatography (HPLC) system coupled to a diode array detector. One microliter sample was injected into an Agilent Zorbax Eclipse Plus C18 4.6 mm × 100 mm, 3.5 µm column (Agilent Technologies, Santa Clara, CA, USA) heated to 30°C. The mobile phase consisted of a 0.05% acetic acid in water (A) and acetonitrile (B). The gradient started as 5% B and followed a linear gradient to 95% B over 8 min. This solvent composition was maintained for 2 min, after which it was changed immediately to 5% B and maintained for 2 min. The elution of the compounds was detected at a wavelength of 270 nm. HPLC data were processed using Chromeleon 7.2.9 software (Thermo Fisher Scientific), and compound concentrations were calculated from authentic calibration standards.

Glucose was quantified on a Dionex Ultimate 3000 HPLC system equipped with a refractive index detector. An Aminex HPX-87H column 7.8 mm × 300 mm (Bio-Rad) with a Micro-Guard Cation H+ guard column 4.6 mm × 30 mm heated to 30°C was injected with 10 µL sample. The mobile phase consisted of 5 mM H_2_SO_4_ with an isocratic flow rate of 0.6 mL/min, which was held for 15 min.

For qualitative confirmation of ABA in supernatant samples, a Dionex 3000 HPLC system connected to an Orbitrap Fusion Mass Spectrometer (Thermo Fisher Scientific, San Jose, CA) was used. The chromatographic separation was achieved using a Waters ACQUITY BEH C18 (10 cm × 2.1 mm, 1.7 μm) column equipped with an ACQUITY BEH C18 guard column kept at 40°C. The mobile phases consisted of MilliQ^®^ water + 0.1% formic acid (A) and acetonitrile + 0.1% formic acid (B). The initial composition was 2% B, held for 0.8 min, followed by a linear gradient till 5% in 3.3 min, and afterward, 100% B was reached in 10 min and held for 1 min before going back to initial conditions. Re-equilibration time was 2.7 min. Flow rate was kept constant at 0.35 mL/min and injection volume was 1 µL. The MS(MS) measurement was done in negative heated electrospray ionization (HESI) mode with a voltage of 2500 V acquiring in full MS/MS spectra (data dependent acquisition-driven MS/MS) in the mass range of 70–1000 Da. The resolution was set at 120 000 for MS and to 30 000 for the MS2. Precursor ions were fragmented by stepped higher energy C-trap dissociation (HCD) using collision energies of 20, 40 and 55.

For qualitative assessment of the presence of ABA-related compounds in unwashed cell pellet extracts, a Dionex 3000 HPLC system connected to an Orbitrap Fusion Mass Spectrometer (Thermo Fisher Scientific, San Jose, CA) equipped with an Agilent Zorbax Eclipse Plus RRHD C18 2.1 × 100 mm, 1.8 µm column (Agilent Technologies, Santa Clara, CA, USA) was used. The column was heated to 35°C, and the mobile phase consisted of a 0.1% formic acid (A) and 0.1% formic acid in acetonitrile (B). The gradient started at 5% B for 0.6 min and followed a linear gradient to 95% B over 13 min. This solvent composition was held for 1.5 min, after which it was changed immediately to 5% B and held until 17.5 min. The sample (1 µL) was passed on to the MS equipped with a HESI source in negative-ion mode with sheath gas set to 50 (a.u.), aux gas to 10 (a.u.) and sweep gas to 2 (a.u.). The cone and probe temperatures were 350°C and 325°C, respectively, and spray voltage was 2750 V. Scan range was 100–1000 m/z, and the resolution was set to 60 000, RF Lens 60%, and AGC target 5.0e4. Precursor ions were fragmented (ddMS2) by HCD-assisted collision energies of 15, 30, 45 and 60 (a.u.).

### Bioreactor cultivation

The preculture was prepared by inoculating strain ST9727 from glycerol stocks into a 250-mL shake flask with 25-mL Y10P20P20 and overnight incubation at 30°C with shaking. The required volume for a starting OD600 of 1.0 was used to inoculate 150-mL of either Y10P20D80 for batch cultivation or Y20P40D5 for fed-batch cultivation in 250-mL Ambr^®^ bioreactors. Dissolved oxygen was maintained at ∼40% or ∼10% by changing the stirring speed ranging from 100 or 1000 to 4000 rpm. The aeration was constant at 150 mL/min. For the conditions with pH control, a set point of 5.5 was chosen and was adjusted by the automatic addition of 2 M H_3_PO_4_ and 2 or 1 M KOH. The automatic addition of antifoam 204 was preprogrammed. Samples were taken automatically every 6 h and immediately frozen until analysis. For the fed-batch cultivation, a d-glucose feed of 500 g/L was used with a constant feed rate of 0.9 mL/h that was initiated 6 h after inoculation.

## Results

### Engineering of *Y. lipolytica* for ABA production

**Figure 2.**
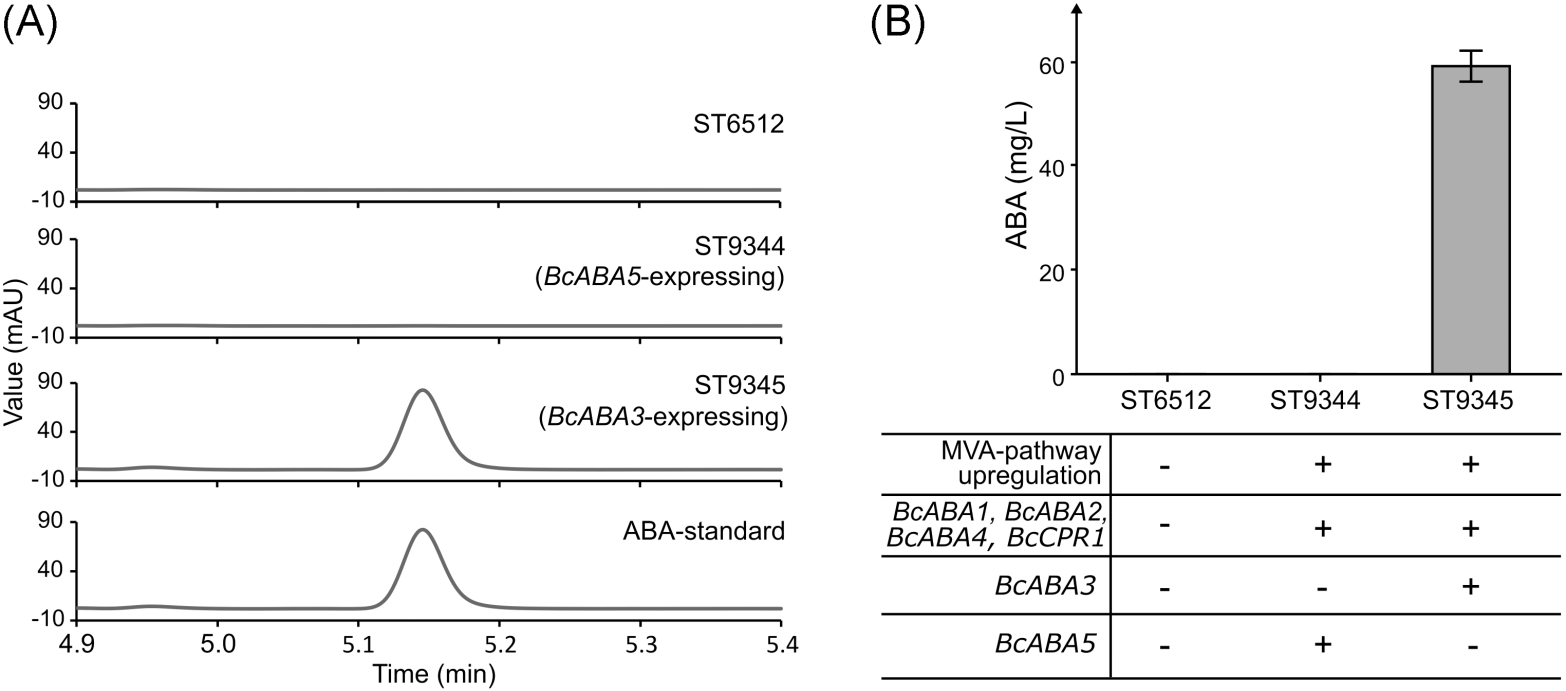
Extracellular ABA production by engineered *Y. lipolytica* strains. **(A)** HPLC chromatograms of the cultivation supernatant from reference strain ST6512 and engineered strains ST9344 (*BcABA5*-expressing strain) and ST9345 (*BcABA3*-expressing strain), and authentic ABA standard. **(B)**ABA concentration in the supernatant from strains ST6512, ST9344 and ST9345. All strains were cultivated for 72 h in Y10P20D80 media in 24-well plates. Titer average and standard deviations for each strain were calculated from cultivation triplicates (*n* = 3).

A strain of *Y. lipolytica* previously modified for increased production of FPP (C_15_-platform strain) was further engineered for ABA production (Arnesen *et al*. 2020). The modifications to improve MVA pathway flux toward FPP in C_15_-platform strain included the expression of the *Salmonella enterica* acetyl-CoA synthetase (*SeACS*), overexpression of the native ATP citrate lyase 1 (*ACL*), 3-hydroxy-3-methylglutaryl coenzyme A reductase (*HMG*), MVA kinase (*ERG12*), isopentyl diphosphate isomerase (*IDI*) and *ERG20*, and downregulation of the native squalene synthase by promoter swapping (*pERG11_SQS*). These modifications were selected from because they have been shown to increase terpene production in various studies (Cao *et al*. 2016, 2017, Yang *et al*. 2016, Kildegaard *et al*. 2017, Huang *et al*. 2018). The performance of the platform strain was validated by producing valencene at 8.4-fold increased levels compared to WT (Liu *et al*. 2019). The genes encoding the ABA biosynthetic pathway enzymes from *B. cinerea* were codon-optimized for *Y. lipolytica* and expressed in the C_15_-platform strain (ST9345, *BcABA3*-expressing strain). This strain expressed *BcABA1*, *BcABA2*, *BcABA3*, *BcABA4*, and a *B. cinerea* cytochrome P450 reductase (*BcCPR1*) that was important for ABA production in *B. cinerea* (Siewers *et al*. 2004). We also constructed a similar strain (ST9344), where *BcABA5* was expressed instead of *BcABA3* (*BcABA5*-expressing strain). *BcABA5* encodes a putative sesquiterpene cyclase from *B. cinerea* ATCC 58025 (Izquierdo-Bueno *et al*. 2018). The two engineered strains were cultivated in deep-well plates, and extracellular and intracellular ABA concentrations were analyzed by HPLC. Only the strain expressing *BcABA3* produced ABA, where 59.2 ± 3.0 mg/L ABA was measured in the broth (Fig. 2A and B). Only negligible amounts of ABA (<0.2 mg/L) were present in the extracts of washed cell pellets. The presence of ABA in the broth was further confirmed by LC-MS analysis, while ABA could not be detected for *BcABA5*-expressing strain or in the parental C_15_-platform strain (Fig. S1, Supporting Information). Furthermore, analysis of unwashed cell pellets from the *BcABA3*-expressing strain showed the presence of multiple compounds that were tentatively identified as ABA precursors or oxidative products based on MS-spectra library comparison (Fig. S2, Supporting Information). One peak was tentatively identified as 1′,4′-*trans*-dihydroxy-α-ionylideneacetic acid, the immediate precursor for ABA. Two other peaks were tentatively identified as xanthoxin and abscisic aldehyde, respectively, which could be oxidated ABA intermediates formed by endogenous enzymes.

### Evaluation of engineering strategies to increase ABA production

**Figure 3.**
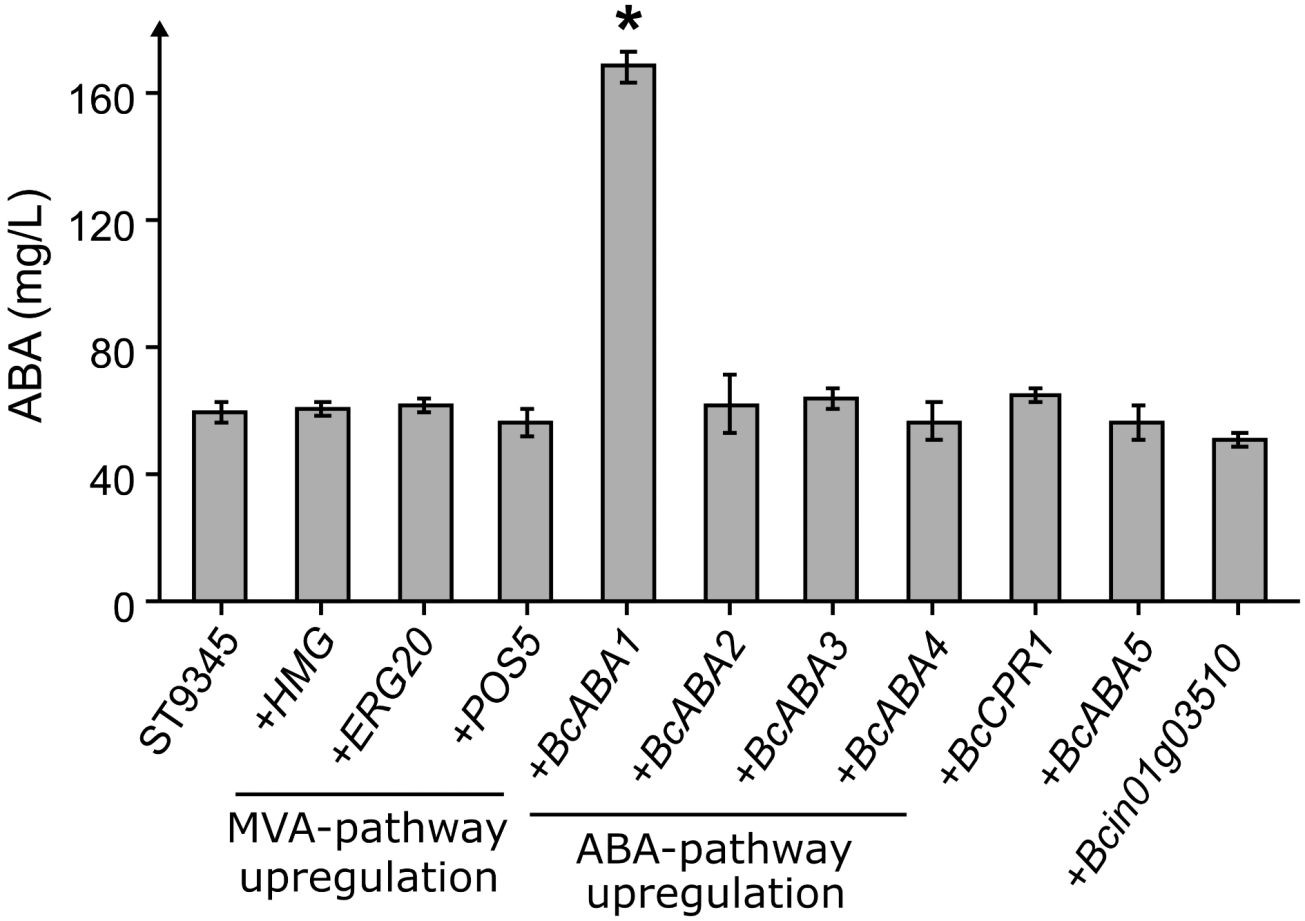
The effect of additional gene expression on ABA titer. The control strain (ST9345) expresses a single copy of the *BcABA1–4* genes. All strains were cultivated for 72 h in Y10P20D80 media in 24-well plates. Titer average and standard deviations for each strain were calculated from cultivation triplicates (*n* = 3). Statistically significant ABA titer increases compared with ST9345 are indicated by an asterisk (*P* < 0.05, *t*-test, critical two-tailed).

To increase ABA production in the *BcABA3*-expressing strain, we tried overexpressing MVA pathway genes *HMG* or *ERG20*, which were reported to increase sesquiterpenoid production in yeast (Ro *et al*. 2006, 2008). We also tested overexpression of *POS5* (*YAli0E17963G*) encoding a putative NAD + kinase to improve the supply of the reduced nicotinamide adenine dinucleotide phosphate (NADPH) redox cofactor, as reported for *S. cerevisiae* (Paramasivan and Mutturi 2017). However, neither overexpression of *HMG*, *ERG20*, nor *POS5* resulted in significant increase of ABA production (Fig. 3). Concurrently, we investigated whether the expression of additional copies of the ABA biosynthetic pathway genes, *BcABA1*,*BcABA2*,*BcABA3* or *BcABA4*, would increase ABA production. Only the expression of the second copy of *BcABA1* affected the ABA titer, which increased 2.8-fold to 168.5 ± 4.8 mg/L (Fig. 3), suggesting that this enzyme performing carboxylation of α-ionylideneethane was limiting the flux through the pathway. Lastly, we investigated whether the expression of genes putatively supporting ABA production could influence the ABA production in *Y. lipolytica*. The expression of an additional copy of the reductase *BcCPR1* did not result in increased ABA production. Furthermore, although BcABA5p has not been shown to catalyze the formation of the ABA precursor α-ionylideneethane, it was still shown to be essential for ABA production in *B. cinerea* (Izquierdo-Bueno *et al*. 2018). Likewise, a gene encoding a putative cytochrome P450 monooxygenase (*Bcin01g03510*) was found to be co-located with *BcABA5* on the genome of *B. cinerea* (Izquierdo-Bueno *et al*. 2018). Therefore, *Bcin01g03510* could also be involved in ABA biosynthesis. However, neither expression of *BcABA5* nor *Bcin01g03510* increased ABA production (Fig. 3).

### Construction of an ABA-overproducing strain

**Figure 4.**
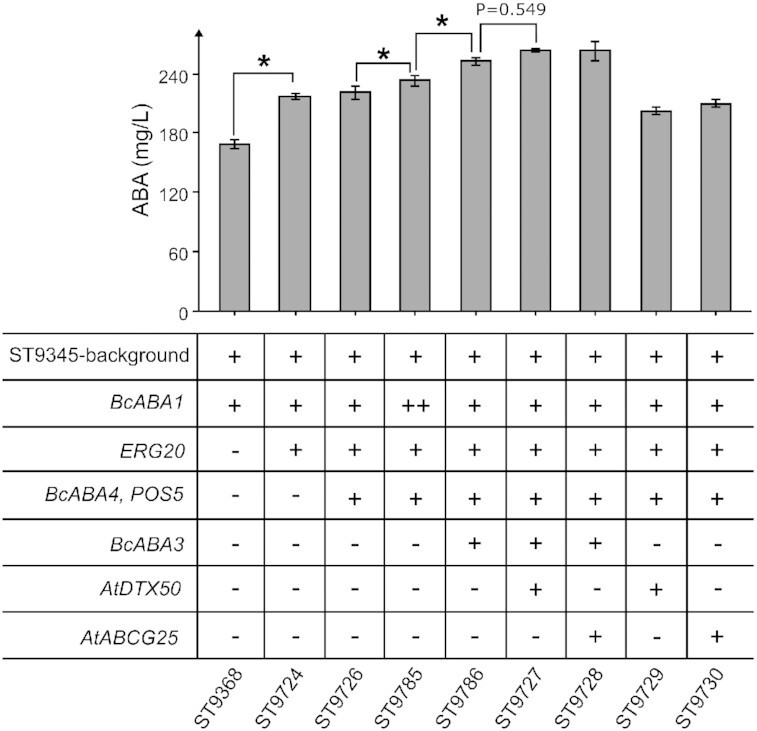
Effect of cumulative gene expression on ABA production in engineered *Y. lipolytica* strains. +, addition of a single gene copy; ++, addition of two gene copies. All strains were cultivated for 72 h in Y10P20D80 media in 24-well plates. Titer average and standard deviations for each strain were calculated from cultivation triplicates (*n* = 3). Statistically significant ABA titer increases are indicated by an asterisk (*P* < 0.05, *t*-test, critical two-tailed).

Upregulation of MVA pathway genes did not have an effect in the *BcABA3*-expressing strain, that only carried a single copy of the pathway. However, expression of an additional copy of the ABA flux limiting *BcABA1* could enable higher MVA flux to positively affect ABA production. Therefore, we expressed another copy of *BcABA1* and *ERG20* (ST9724) in the *BcABA3*-expressing strain. This resulted in a titer increase to 217.0 ± 3.1 mg/L ABA, a 29% increase over only expressing another copy of *BcABA1* (Fig. 4). Next, we expressed *POS5* and another copy of *BcABA4* (ST9726). However, no significant increase in ABA production was achieved in this strain compared to its parental strain (Fig. 4). We then attempted to further improve the flux through early-stage ABA biosynthesis by expressing either a third copy of *BcABA1* (ST9785) or a second copy of *BcABA3* (ST9786) in ST9726. The expression of a third copy of *BcABA1* increased ABA production by 5% to 232.6 ± 5.2 mg/L, while the expression of a second copy of *BcABA3* led to a greater increase by 15% to 252.6 ± 4.1 mg/L ABA (Fig. 4). Although the engineered strains were able to export ABA to the supernatant, we wanted to test is the secretion could be improved further by expression of plant transporters involved in ABA transport. Providing the engineered strain with additional ABA transporters could alleviate cellular stress, which has been found to occur during the production of other sesquiterpenoids in yeast, even though these compounds also were secreted by the yeast cells (Ro *et al*. 2008). We therefore expressed two transporters from *A. thaliana*, AtDTX50p and AtABCG25p, have been implicated in ABA export in plant cells and heterologous systems, in our strains (Kuromori *et al*. 2010, Zhang *et al*. 2014). Expression of *AtDTX50* alongside a second copy of *BcABA3* (ST9727) in ST9726 resulted in 263.5 ± 1.8 mg/L and 9.1 ± 0.1 mg/g DCW ABA, while expression of *AtABCG25* alongside a second copy of *BcABA3* (ST9728) in ST9726 resulted in 262.8 ± 9.5 mg/L and 8.8 ± 0.4 mg/g DCW ABA. These titers were not significantly increased (*P *> 0.05) compared to ST9786 expressing two copies *BcABA3* without heterologous transporters (Fig. 4). Expression of *AtDTX50* or *AtABCG25* in ST9726 without an additional copy of *BcABA3* did not positively impact ABA production.

### Investigation of growth and ABA production profile of ABA overproducer

**Figure 5.**
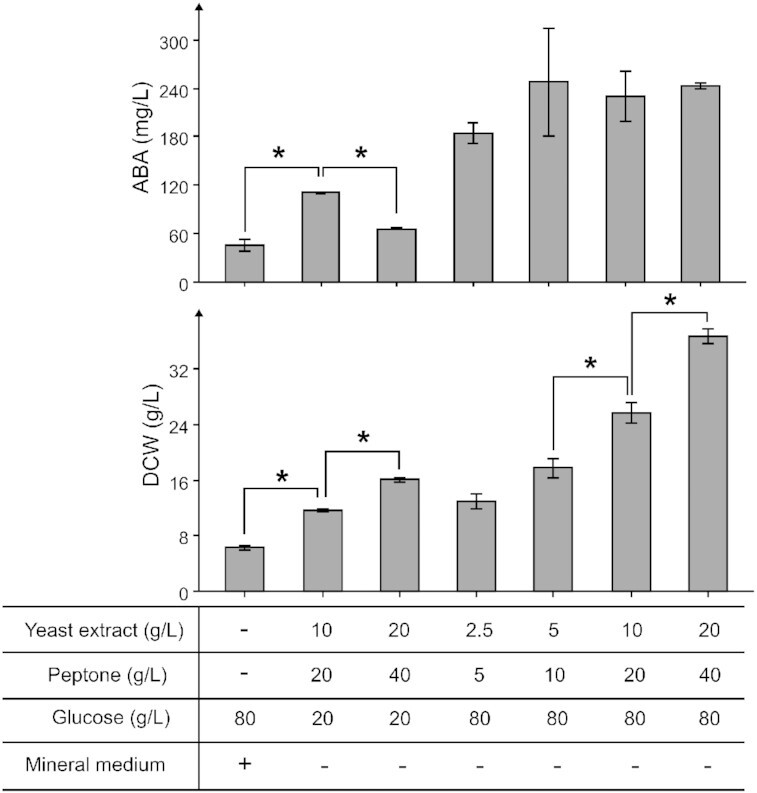
The effects of media composition on ABA and biomass accumulation of ST9727 during small-scale cultivation. ST9727 was cultivated for 72 h with variable media compositions in 24-well plates. Titer average and standard deviations for each strain were calculated from cultivation triplicates (*n* = 3). An asterisk indicates statistically significant ABA titer and biomass differences (*P* < 0.05, *t*-test, critical two-tailed).

**Figure 6.**
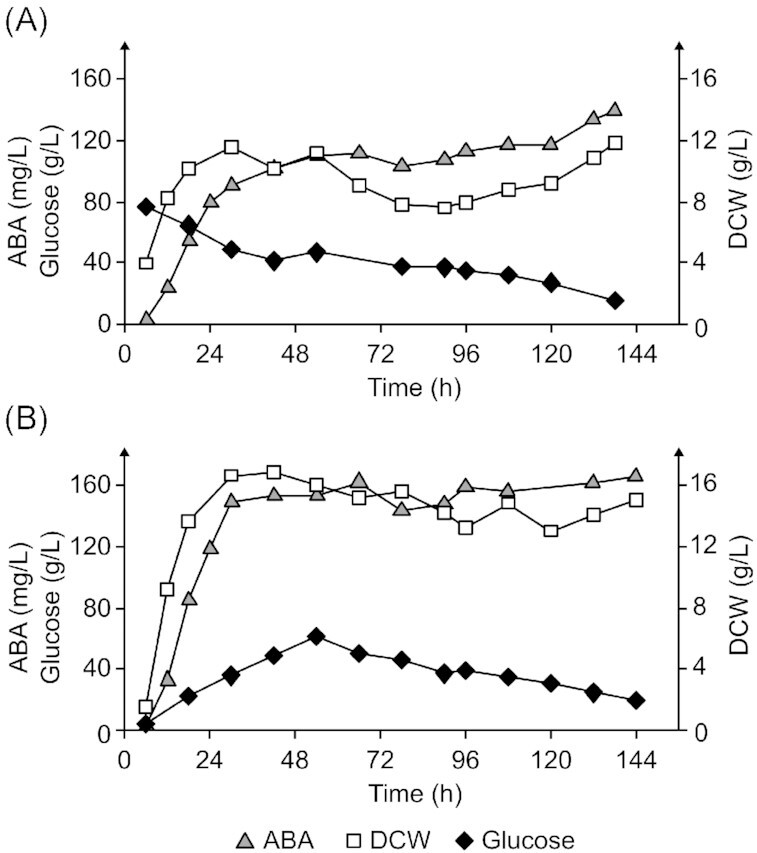
Time course of ABA, DCW and glucose concentration dynamics during cultivation of ST9727 from representative bioreactors during **(A)** batch (Bioreactor 1) and**(B)** fed-batch cultivation (Bioreactor 4). The other replicates are shown in Fig. S3 (Supporting Information).

Following genetic engineering, we sought to investigate the growth and ABA production profile of ST9727 during small-scale cultivation in different media. Rich media based on YPD has commonly been used for terpenoid production in *Y. lipolytica* (Larroude *et al*. 2018, Liu *et al*. 2020). However, defined media has previously been used to reach high titers of resveratrol in *Y. lipolytica* (Sáez-Sáez *et al*. 2020). Therefore, we tested standard Y10P20D20 against mineral media. Y10P20D20-media proved superior to mineral media for both ABA and biomass accumulation (Fig. 5). Furthermore, increasing the concentration 2-fold of YP by using Y20P40D20 lowered ABA production compared to Y10P20D20 but increased biomass accumulation. Nitrogen limitation can be used to increase the lipid biosynthesis in *Y. lipolytica* (Bellou *et al*. 2016, Kerkhoven *et al*. 2016). We therefore tested complex media with higher C/N ratios. No differences in ABA titers were found between the conditions Y5P10D80, Y10P20D80, and Y20P40D80 (Fig. 5). However, biomass increased in response to higher levels of YP in Y5P10D80, Y10P20D80, and Y20P40D80, respectively. We then investigated the temporal growth and ABA production profile of ST9727 during batch cultivation with Y10P20D80 in 250-mL Ambr^®^ bioreactors with DO set to >40%, pH control at 5.5 and a minimum stirring speed of 1000 rpm in triplicate (Fig. 6A; Fig. S3A and B, Supporting Information). The biomass concentration increased until ∼30 h of cultivation, while the ABA titer increased until ∼54 h. Interestingly, growth halted at 30 h despite the glucose concentration still being at 49.4 g/L. Phosphoric acid was automatically added during the first 18 h of cultivation to prevent a rise in pH, which may have been caused by ammonia release from the degradation of amino acids and peptides (Fig. S4A, Supporting Information). Furthermore, a decrease in biomass concentration from 11.3 ± 0.6 to 7.9 ± 0.1 g/L (*P* < 0.05, *t*-test, critical two-tailed) was observed from 30 to 78 h, whereafter biomass trended toward an increase to 10.5 ± 1.1 g/L at 138 h (*P* = 0.059, *t*-test, critical two-tailed). Only 133.6 ± 4.6 mg/L ABA was achieved after 138 h of cultivation in 250-mL Ambr^®^ reactors, considerably less than in 72 h of small-scale cultivation. Therefore, the current cultivation conditions seem to impose limitations on the growth and ABA production of ST9727. However, a biomass-specific yield of 12.8 ± 0.9 mg/g DCW was achieved, which was a significant increase over the 9.1 ± 0.1 mg/g DCW achieved during small-scale cultivation (*P* < 0.05, *t*-test, critical two-tailed).

Concurrent with the batch cultivations, we tested a fed-batch cultivation procedure with Y20P40D5 as starting media and a constant glucose feed since such methods have previously been used to obtain high terpenoid titers in *Y. lipolytica* (Larroude *et al*. 2018, Tramontin *et al*. 2019). Such procedures could induce nitrogen limitation after biomass accumulation, which may increase acetyl-CoA and terpenoid accumulation. Otherwise, the fed-batch cultivations had the same conditions described earlier. However, after ∼49 h, glucose accumulated to ∼60 g/L, so we stopped the glucose feed. By the end of cultivation, 167.1 ± 2.9 mg/L and 10.6 ± 0.4 mg/g DCW ABAs were achieved (Fig. 6B; Fig. S3C and D, Supporting Information).

**Figure 7.**
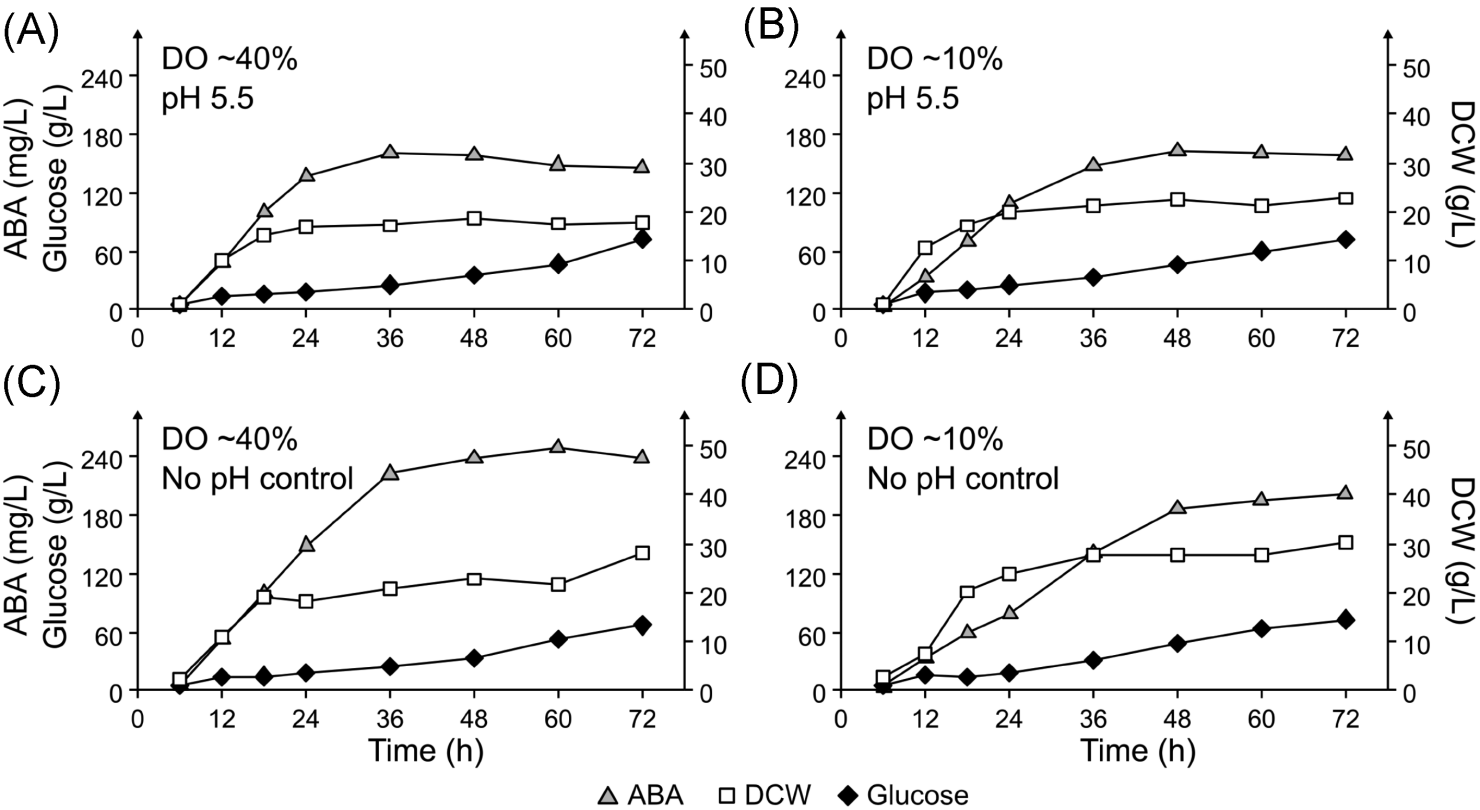
Time course of ABA, DCW and glucose concentration dynamics during fed-batch cultivation of ST9727 from representative bioreactors under various conditions. **(A)** DO ∼40% and pH control set to 5.5 (Bioreactor A). **(B)** DO ∼10% and pH control set to 5.5 (Bioreactor C). **(C)** DO ∼40% and no pH control (Bioreactor E). **(D)** DO ∼10% and no pH control (Bioreactor G). The other replicates are shown in Fig. S6 (Supporting Information).

It was unexpected that the ABA titer in the bioreactors was much lower than that in small-scale cultivations. We speculated whether these differences could be due to higher oxygenation or higher pH in bioreactors. Therefore, we cultivated strain ST9727 in fed batch in bioreactors at two preset dissolved oxygen DO levels (∼10% or ∼40%) and with pH control at 5.5 or without pH control, where pH would drop during the cultivation, as for small-scale cultivations. The four combinations of conditions were as follows: DO ∼40% and pH control set to 5.5 (Bioreactors A and B), DO ∼10% and pH control set to 5.5 (Bioreactors C and D), DO ∼40% and without pH control (Bioreactors E and F), and DO ∼10% and without pH control (Bioreactors G and H) (Fig. 7; Figs S6–S9, Supporting Information). DO levels were controlled by changing stirring speed, where the minimum was set to 100 rpm. At 60 h, the ABA titers and biomass-specific yields were 147.4 ± 1.3 mg/L and 8.9 ± 0.7 mg/g DCW for DO ∼40% with pH 5.5, 161.4 ± 1.5 mg/L and 7.1 ± 0.5 mg/g DCW for DO ∼10% with pH 5.5, 243.5 ± 6.7 mg/L and 10.8 ± 1.0 mg/g DCW for DO ∼40% without pH control, and 198.6 mg/L and 7.2 ± 0.3 mg/g DCW for DO ∼10% without pH control. It seems that the cultivations without pH control performed better than those with pH 5.5, and that DO at ∼40% was superior to DO ∼10% during cultivation without pH control. Seemingly, the bioreactor cultivations with DO ∼40% without pH control performed comparably to 72 h small-scale cultivations with Y10P20D80.

## Discussion

The highest reported titers (up to 2 g/L) of ABA are based on cultivation of the filamentous fungi *B. cinerea* (Gong *et al*. 2014, Ding *et al*. 2016, Shi *et al*. 2017). The engineered *Y. lipolytica* strain produced 263.5 ± 1.8 mg/L ABA in small-scale cultivation, which is a considerable improvement over previous titers of 8 and 11 mg/L reported for engineered strains of *A. oryzae* and *S. cerevisiae*, respectively (Otto *et al*. 2019, Takino *et al*. 2019). Therefore, *Y. lipolytica* is a promising host for heterologous ABA production, although further titer improvements are necessary before such engineered strains can be used industrially. Although the ABA biosynthetic pathway consisting of *BcABA1*, *BcABA2*,*BcABA3* and *BcABA4* from *B. cinerea* has been characterized, another report indicates that the putative sesquiterpenoid cyclase *BcABA5* gene is also involved in ABA biosynthesis in *B. cinerea* (Izquierdo-Bueno *et al*. 2018). However, expression of *BcABA5* together with *BcABA1*, *BcABA2*,*BcABA4* and *BcCPR1* in *Y. lipolytica* did not result in ABA production, suggesting that BcABA5p does not form the ABA precursor α-ionylideneethane, which is consistent with previous results (Otto *et al*. 2019). Therefore, it remains unclear how *BcABA5* may be involved in ABA biosynthesis in *B. cinerea*. It has been demonstrated that fungal biosynthetic pathways and gene clusters can exert regulatory effects on each other, even if no metabolites are shared between the pathways (Bergmann *et al*. 2010, Wiemann *et al*. 2012, Hidalgo *et al*. 2014). Interestingly, it was previously found that multicopy expression of *BcABA1* and, to a lesser extent, *BcABA3* increased ABA production in *S. cerevisiae* (Otto *et al*. 2019). This is consistent with our results, where expression of a second *BcABA1* copy increased ABA production. The ABA production increase in the engineered *Y. lipolytica* strain by multicopy *BcABA3* expression was dependent on sufficient flux through the carboxylation step of α-ionylideneethane mediated by BcABA1p. We detected a number of ABA-related compounds in engineered *Y. lipolytica*, which indicates insufficient activity of some enzymatic steps or side reactions leading to the degradation of intermediates. This information can be used to design strategies for further strain improvement. Methods like nuclear magnetic resonance spectroscopy may be needed to determine the structure of the ABA-related compounds accurately.

Although ABA was efficiently secreted to the media, previous literature has demonstrated that heterologous sesquiterpenoid production can result in cellular stress (Ro *et al*. 2008). Likewise, increasing ABA export could exert a ‘pulling’ effect on the ABA biosynthetic pathway leading to improved flux toward ABA. However, the expression of heterologous ABA transporters did not increase ABA production, which indicates that the native yeast transporters sufficiently export the majority of ABA at the current production levels. Similarly, ABA was also secreted to the extracellular matrix by engineered *S. cerevisiae* and the natural producer *B. cinerea*, which indicates the presence of membrane transporters with shared functionalities in these species (Gong *et al*. 2014, Ding *et al*. 2016, Otto *et al*. 2019). Interestingly, it was found that ABA production occurred mainly during the first 16 h of cultivation in engineered *S. cerevisiae*, which is similar to engineered *Y. lipolytica* that produces ABA mainly during the early cultivation period. This is contrasted by the production profile of *B. cinerea* where ABA seems to accumulate continuously during cultivation and during the stationary phase (Gong *et al*. 2014, Ding *et al*. 2016).

Surprisingly, cultivation of the highly productive strain ST9727 in 250-mL Ambr^®^ bioreactors resulted in generally lower ABA and biomass concentrations than in small-scale cultivations. This suggests that certain conditions of the current bioreactor cultivation inhibit the performance of ST9727. We found that allowing the pH to fluctuate seemingly improved ST9727 performance, but these results were at best comparable to the small-scale cultivations. Interestingly, it was previously shown that an engineered strain of *Y. lipolytica* produced higher astaxanthin titers during fed-batch shake flask cultivations than in fed-batch cultivations in 3-L bioreactors (Ma *et al*. 2021).

The engineering of the yeast chassis could cause increased susceptibility to stressors. It was previously shown that increasing the gene copy numbers of a heterologous cytochrome P450 and CPR in *S. cerevisiae* engineered for protopanaxadiol production resulted in a proportional increase in formation of reactive oxygen species (ROS) and reduced growth on YPD supplemented with dodecane (Zhao *et al*. 2016). Inefficient electron transfer from NADPH mediated by cytochromes P450 and their reductases can generate ROS (Zangar *et al*. 2004). Likewise, *in vitro* studies demonstrate that ROS generation increases with higher CPR amounts (Manoj *et al*. 2010). Therefore, the high constitutive expression of heterologous cytochromes P450 and CPRs in ST9727 could lead to ROS formation and decreased resistance to other sources of cellular stress. This may contribute to the poor performance of ST9727 under stressful conditions particular to bioreactor cultivation. Nevertheless, we demonstrate that engineering of *Y. lipolytica* led to the highest ABA production reported for a heterologous host. Further engineering of the yeast chassis and cultivation procedures is necessary before heterologous ABA production becomes a viable industrial option.

## Acknowledgments

The authors thank the ARS Culture Collection, NCAUR, USA, for providing the W29 *Y. lipolytica* strain Y-63764. The authors thank Marc Cernuda Pastor for help with the plasmid construction and strain transformation, and Lars Boje Petersen for help with the HPLC analysis. The authors also thank Christoffer Knudsen, Drude Mangaard, Jacqueline Medina and Paulina Rubaszka for assistance during the bioreactor cultivations. The graphical abstract was created with BioRender.com.

## Supplementary Material

foac015_Supplemental_File
