# Supplementary material for: Production of abscisic acid in the oleaginous yeast Yarrowia lipolytica
Source: FEMS Yeast Res. 2022 Mar 11;22(1):foac015. doi: 10.1093/femsyr/foac015 (PMC8992728; doi:10.1093/femsyr/foac015)
Supplement: foac015_Supplemental_File [file foac015_supplemental_file.docx]

## Supplementary figures


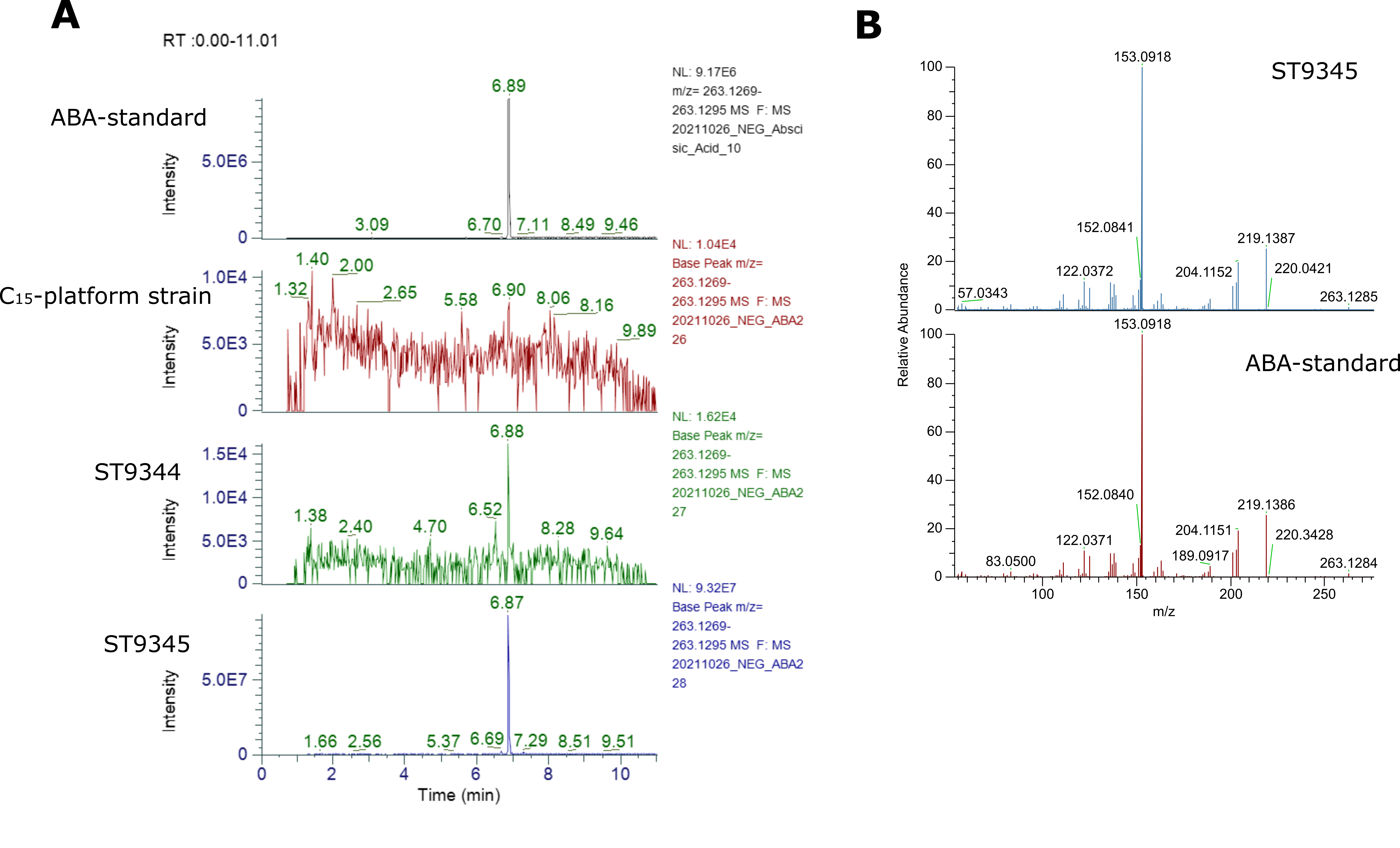


Figure 1: **(A)** extracted ion-chromatogram from LC-MS analysis of supernatant from C15-platform strain, ST9344, ST9345, and authentic ABA-standard. **(B)** Comparison of MS-fragmentation pattern of ABA from ST9345 and authentic standard.


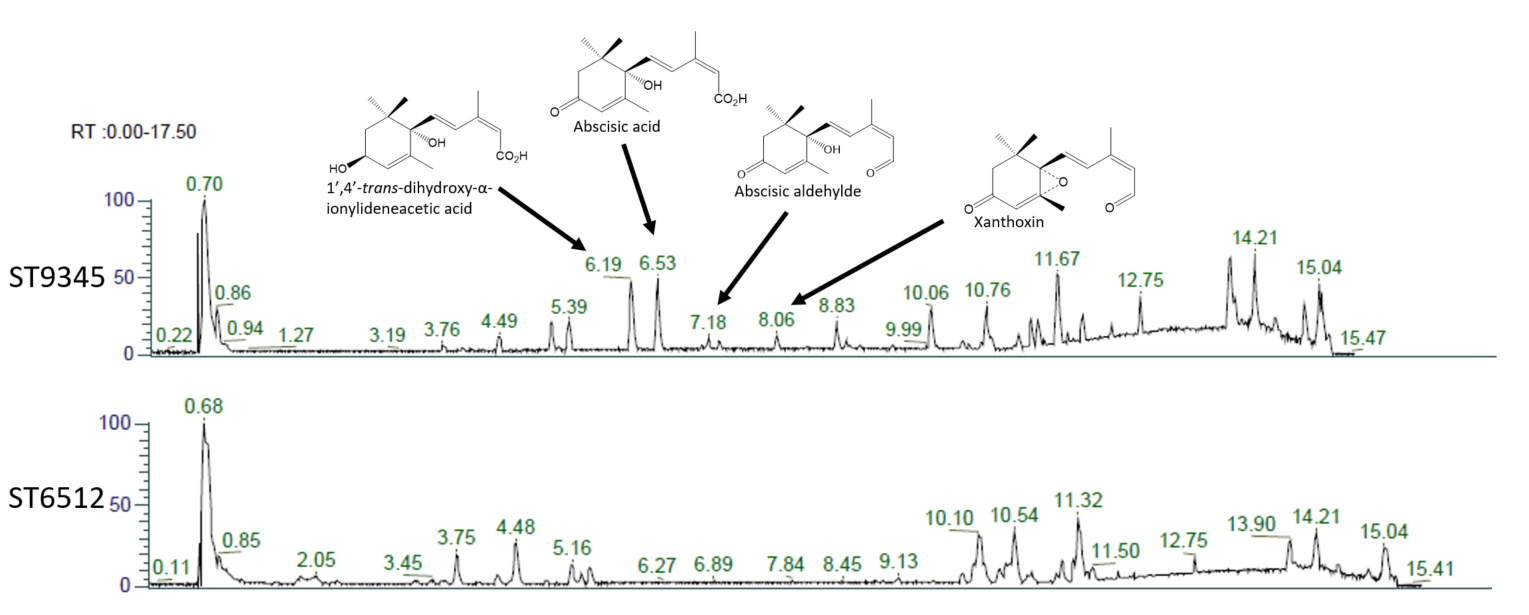


Figure 2: LC-MS chromatograms for ST9345 and ST6512 with ABA with tentatively identified ABA-intermediates and -oxidative products marked.


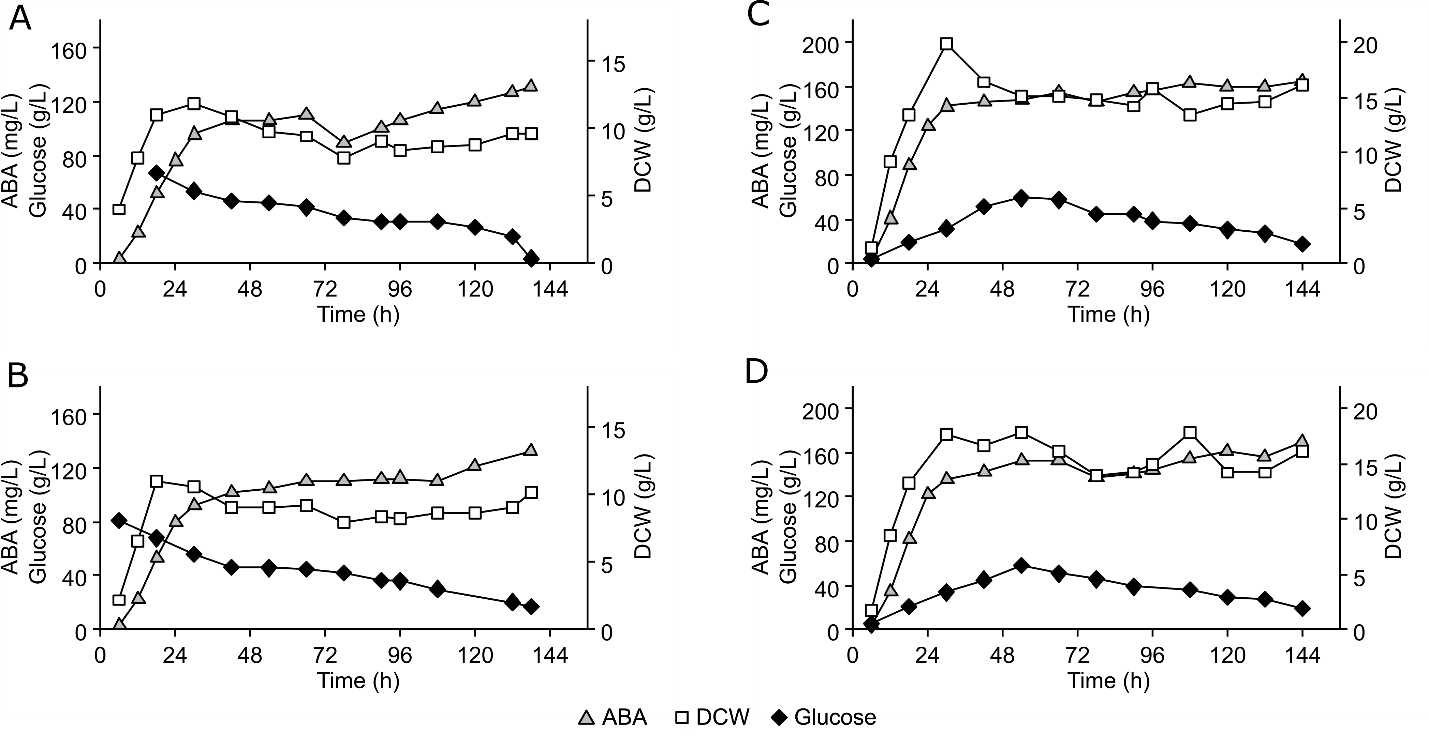


Figure 3: Time-course of ABA, dry cell weight (DCW), and glucose concentration during cultivation of ST9727 from during **(A)** batch fermentation (Bioreactor 2), **(B)** batch fermentation (Bioreactor 3), **(C)** fed-batch fermentation (Bioreactor 5), and **(D)** fed-batch fermentation (Bioreactor 6)


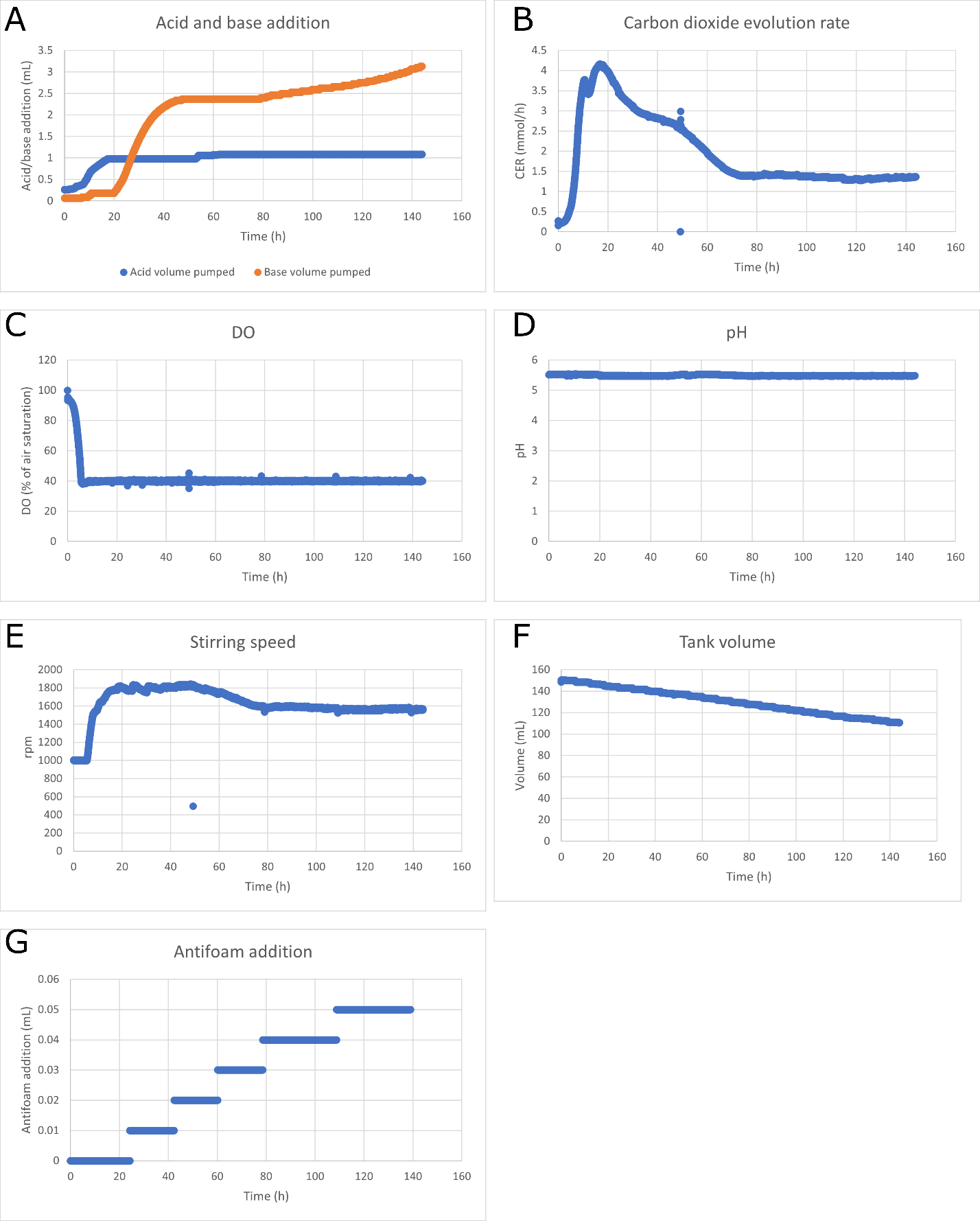


Figure 4: Online measurements of fermentation parameter kinetics for bioreactor 1. **(A)** Acid and base addition. **(B)** Carbon dioxide evolution rate (CER). **(C)** Dissolved oxygen (DO). **(D)** pH. **(E)** Stirring speed. **(F)** Total tank volume. **(G)** Antifoam addition.


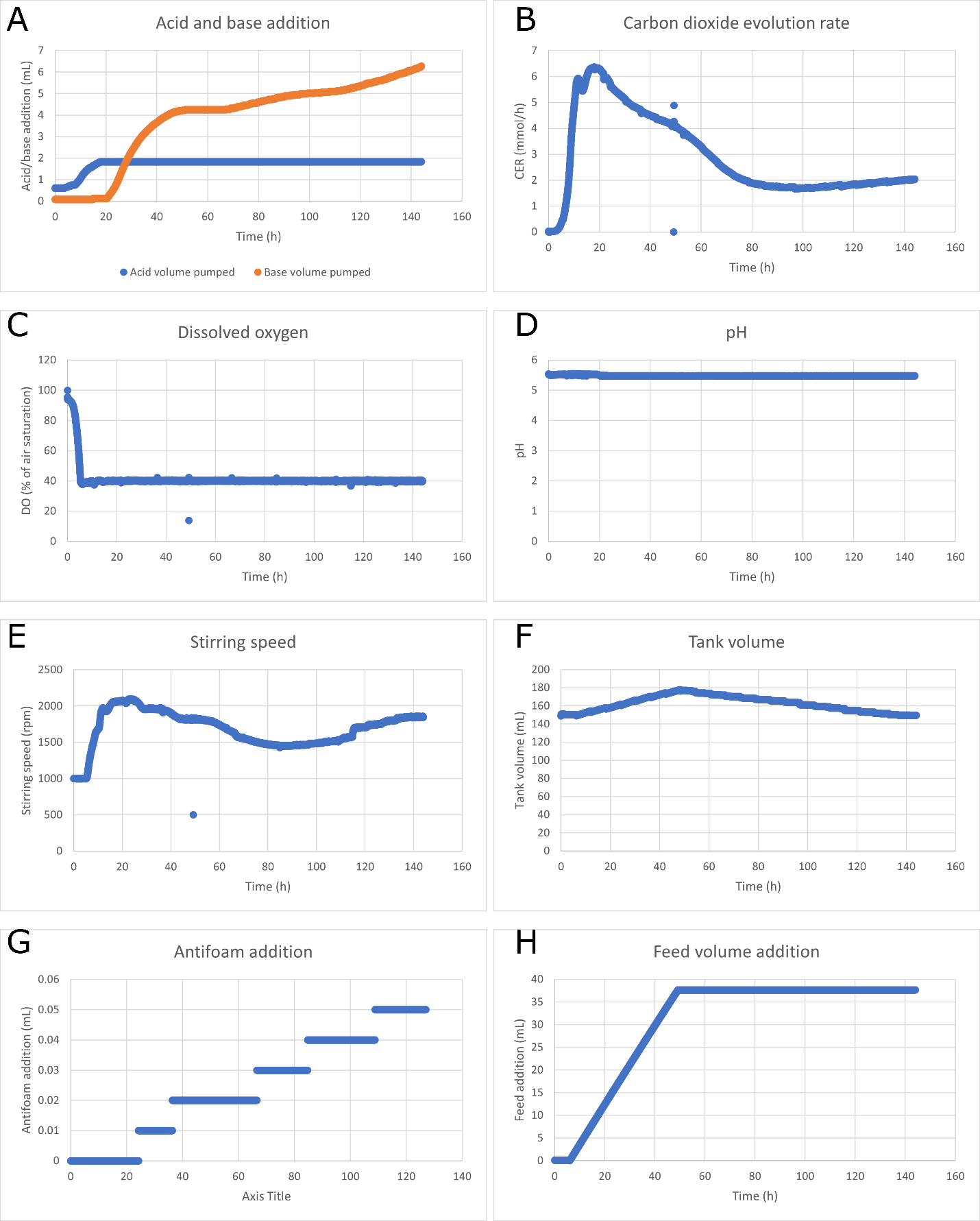


Figure 5: Online measurements of fermentation parameter kinetics for bioreactor 4. **(A)** Acid and base addition. **(B)** CER. **(C)** DO. **(D)** pH. **(E)** Stirring speed. **(F)** Total tank volume. **(G)** Antifoam addition. **(H)** Feed addition.


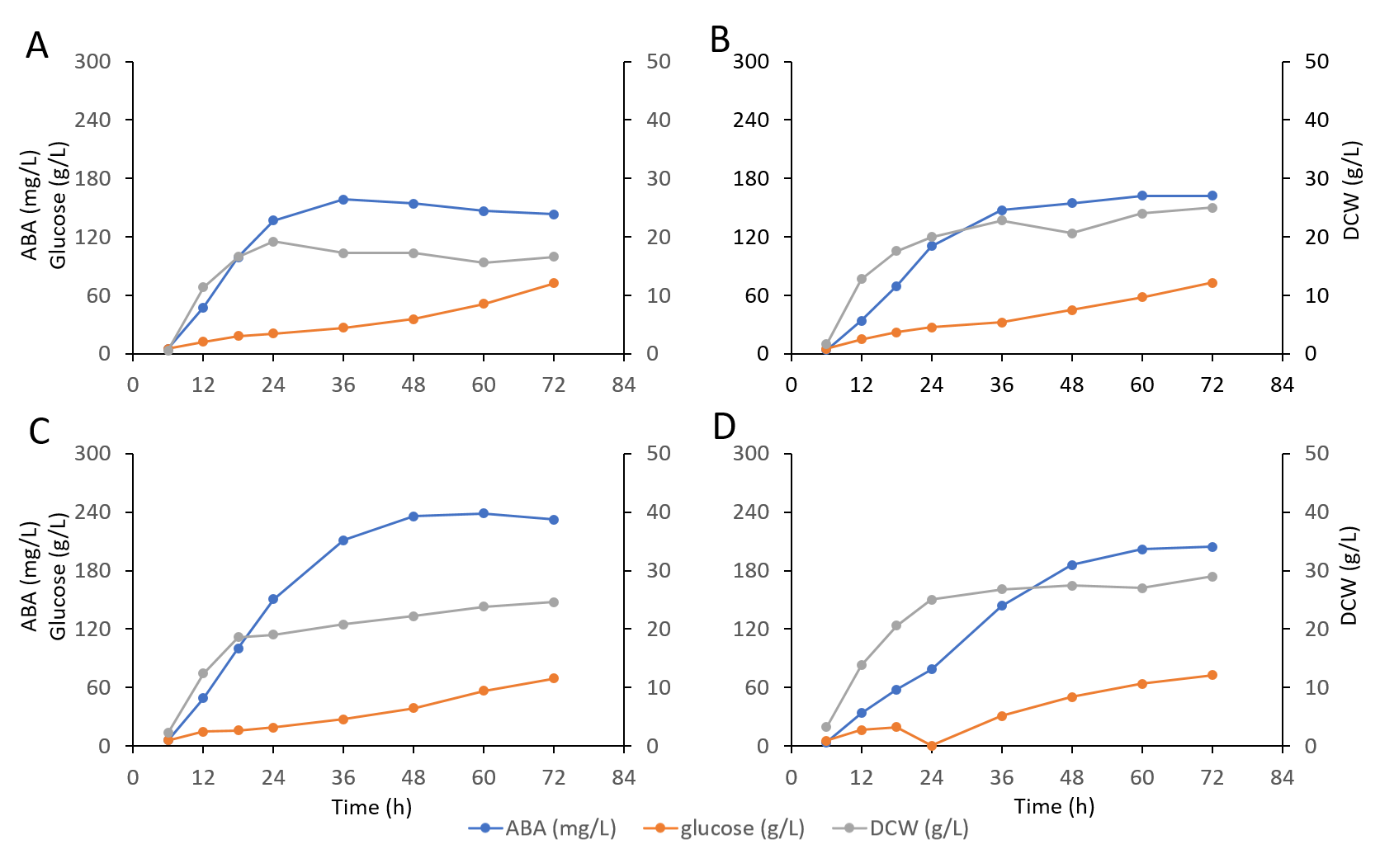


Figure 6: Time-course of ABA, dry cell weight (DCW), and glucose concentration dynamics during fed-batch cultivation of ST9727 from representative bioreactors under various conditions. **(A)** DO >40% and pH-control set to 5.5 (Bioreactor B). **(B)** DO >10% and pH-control set to 5.5 (Bioreactor D). **(C)** DO >40% and no pH-control (Bioreactor F). **(B)** DO >10% and no pH-control (Bioreactor H).


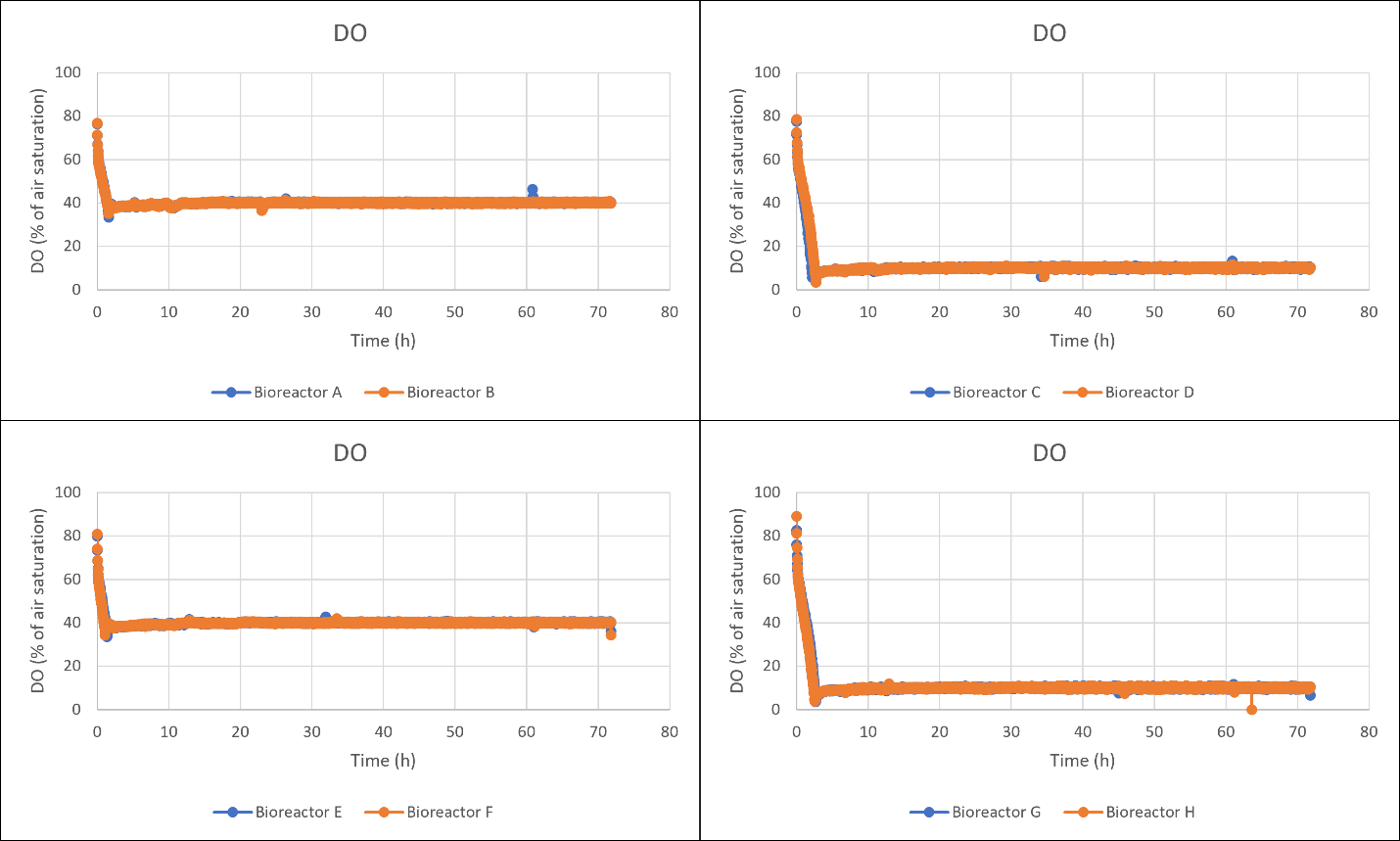


Figure 7: Dissolved oxygen measurements for bioreactors A-H.


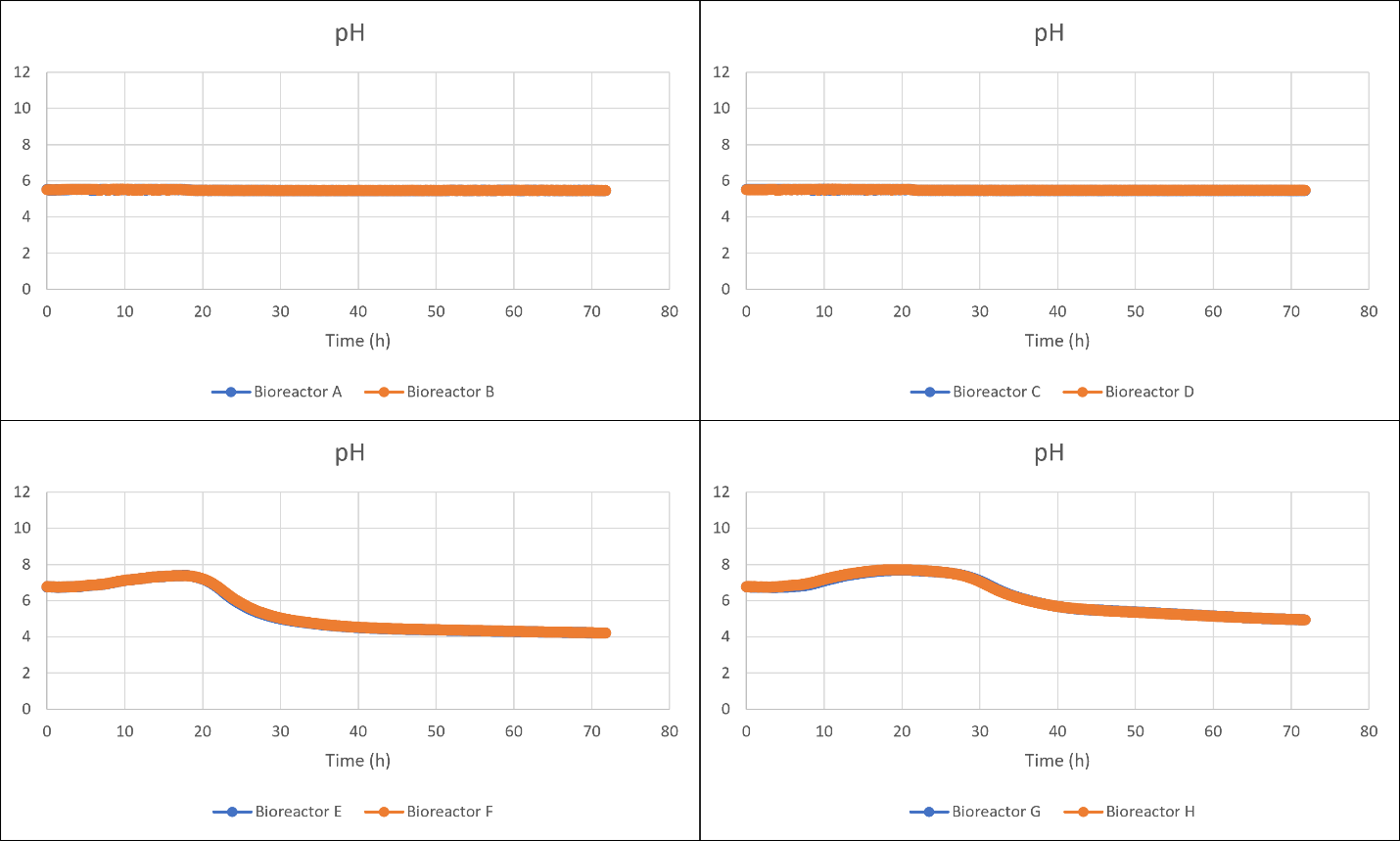


Figure 8: pH measurements for bioreactors A-H.


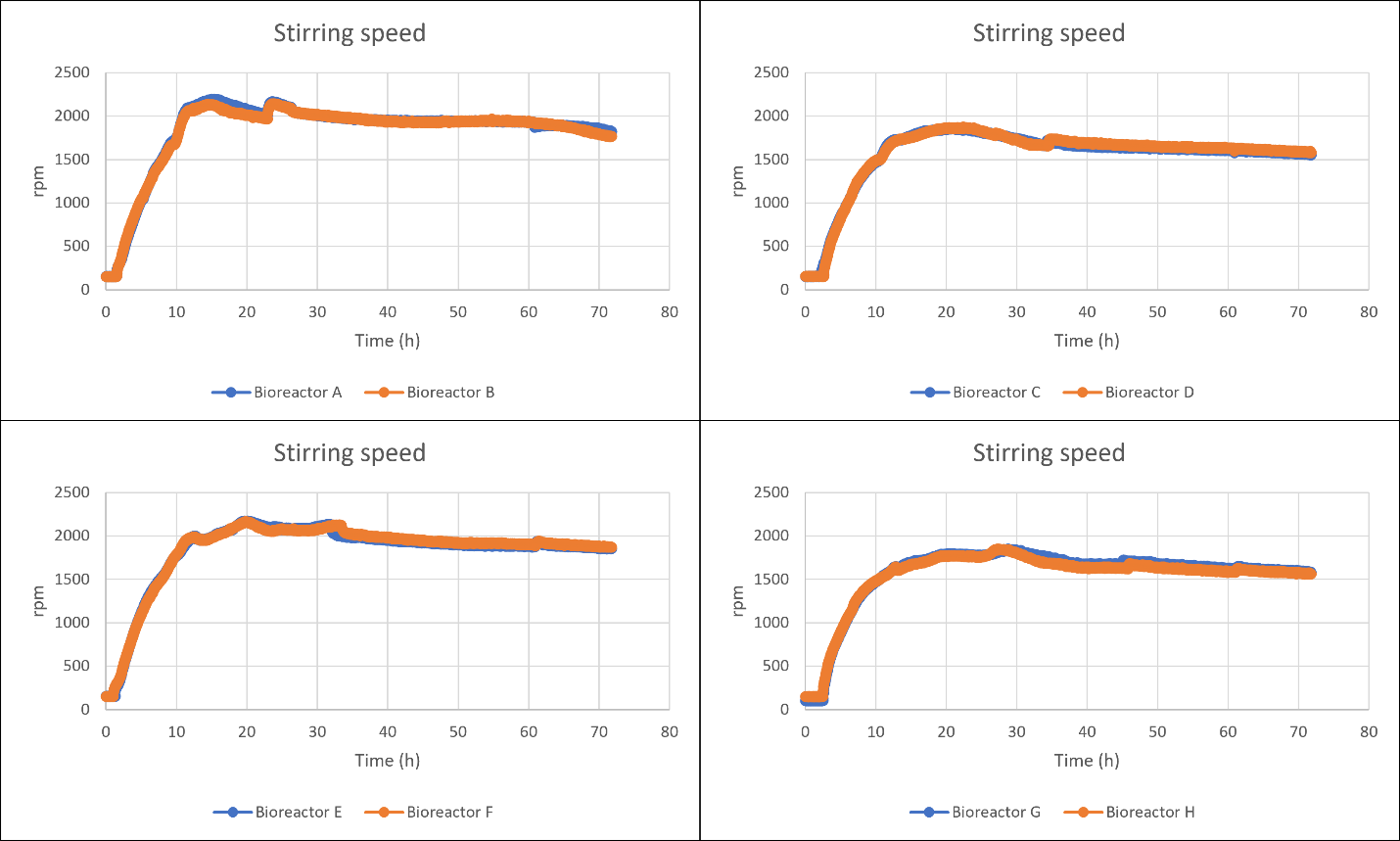


Figure 9: Stirring speed for bioreactor A-H.

**Supplementary table 1.** Strains used in this study

| Strain ID | Genotypes | Parent strain | Plasmid/ Biobricks | Reference |
| --- | --- | --- | --- | --- |
| ST3683 | mus51∆, nugm -Htg2, ndh 2i, lys11-, leu2-, ura3-, MatB |  |  | (Angerer *et al.* 2014) |
| ST4842 | MATa |  |  | *Y. lipolytica* W29 (MatA, ATCC©20460TM) strain Y-63746 from the ARS culture collection. |
| ST6512 | ST4842 ku70∆::PrTEF1->Cas9-TTef12::PrGPD->DsdA-TLip2 |  |  | (Marella *et al.* 2020) |
| ST9149 | ST6512 IntC_2-HMG1<-PrGPD-PrTefInt->ERG12 pCfB8823 IntC_3-SeACS<-PrGPD-PrTefInt->YlACL1 IntD_1-IDI1<-PrGPD-PrTefInt->ERG20 pERG11::pSQS1 |  |  | (Arnesen *et al.* 2020) |
| ST9205 | ST9149 IntE_4-BcCPR1<-PrGPD-TefInt->BcABA5 | ST9149 | pCfB6638 (pNat-YLgRNA2_IntE_4):pCfB8947 (IntE_4-BcCPR1<-PrGPD-TefInt->BcABA5) | This study |
| ST9255 | ST9205 IntE_1-BcABA1<-PrGPD-TefInt->BcABA2 | ST9205 | pCfB8858 (pHphM-YLgRNA2_IntE_1):pCfB8948 (IntE_1-BcABA1<-PrGPD-TefInt->BcABA2) | This study |
| ST9344 | ST9255 IntF_3-TefInt->BcABA4 | ST9255 | pBP8003 (pNat-YLgRNA4-IntF_3):pCfB9109 (IntF_3-TefInt->BcABA4) | This study |
| ST9206 | ST9149 IntE_4-BcCPR1<-PrGPD-TefInt->BcABA3 | ST9149 | pCfB6638 (pNat-YLgRNA2_IntE_4):pCfB8946 (IntE_4-BcCPR1<-PrGPD-TefInt->BcABA3) | This study |
| ST9256 | ST9206 IntE_1-BcABA1<-PrGPD-TefInt->BcABA2 | ST9206 | pCfB8858 (pHphM-YLgRNA2_IntE_1):pCfB8948 (IntE_1-BcABA1<-PrGPD-TefInt->BcABA2) | This study |
| ST9345 | ST9256 IntF_3-TefInt->BcABA4 | ST9256 | pBP8003 (pNat-YLgRNA4-IntF_3):pCfB9109 (IntF_3-TefInt->BcABA4) | This study |
| ST9366 | ST9345 IntE_3-TefInt->HMG | ST9345 | pCfB6637 (pNat-YLgRNA3_IntE_3):pCfB9122 (IntE_3-TefInt->HMG) | This study |
| ST9367 | ST9345 IntE_3-TefInt->BcABA5 | ST9345 | pCfB6637 (pNat-YLgRNA3_IntE_3):pCfB9123 (IntE_3-TefInt->BcABA5) | This study |
| ST9368 | ST9345 IntE_3-TefInt->BcABA1 | ST9345 | pCfB6637 (pNat-YLgRNA3_IntE_3):pCfB9124 (IntE_3-TefInt->BcABA1) | This study |
| ST9369 | ST9345 IntE_3-TefInt->BcABA2 | ST9345 | pCfB6637 (pNat-YLgRNA3_IntE_3):pCfB9125 (IntE_3-TefInt->BcABA2) | This study |
| ST9370 | ST9345 IntE_3-TefInt->BcCPR1 | ST9345 | pCfB6637 (pNat-YLgRNA3_IntE_3):pCfB9126 (IntE_3-TefInt->BcCPR1) | This study |
| ST9371 | ST9345 IntE_3-TefInt->Bcin01g03510 | ST9345 | pCfB6637 (pNat-YLgRNA3_IntE_3):pCfB9127 (IntE_3-TefInt->Bcin01g03510) | This study |
| ST9372 | ST9345 IntE_3-TefInt->ERG20 | ST9345 | pCfB6637 (pNat-YLgRNA3_IntE_3):pCfB9128 (IntE_3-TefInt->ERG20) | This study |
| ST9373 | ST9345 IntE_3-TefInt->BcABA4 | ST9345 | pCfB6637 (pNat-YLgRNA3_IntE_3):pCfB9108 (IntE_3-TefInt->BcABA4) | This study |
| ST9374 | ST9345 IntE_3-PrTefInt->POS5_YAli0e17963G | ST9345 | pCfB6637 (pNat-YLgRNA3_IntE_3):pCfB8870 (IntE_3-PrTefInt->POS5_YAli0e17963G) | This study |
| ST9502 | ST9345 IntE_3-TefInt->BcABA3 | ST9345 | pCfB6637 (pNat-YLgRNA3_IntE_3):pCfB9192 (IntE_3-TefInt->BcABA3) | This study |
| ST9724 | ST9345 IntE_3-ERG20<-GPD-tefInt->BcABA1 | ST9345 | pCfB6637 (pNat-YLgRNA3_IntE_3):pCfB9399 (IntE_3-ERG20<-GPD-tefInt->BcABA1) | This study |
| ST9726 | ST9724 IntA_1-POS5<-GPD-tefInt->BcABA4_PrExp->Hph | ST9724 | pCfB9400 (IntA_1-POS5<-GPD-tefInt->BcABA4_PrExp->Hph) | This study |
| ST9785 | ST9726 pIntE_2-Nat-Tefint->BcABA1 | ST9726 | pCfB9666 (pIntE_2-Nat-Tefint->BcABA1) | This study |
| ST9786 | ST9726 pIntE2-Nat-Tefint->BcABA3 | ST9726 | pCfB9679 (pIntE_2-Nat-Tefint->BcABA3) | This study |
| ST9729 | ST9726 tefInt->DTX50_Prtefint->Nat | ST9726 | pCfB9403 (IntE_2-tefInt->DTX50_Prtefint->Nat) | This study |
| ST9730 | ST9726 AtABCG25_Prtefint->Nat | ST9726 | pCfB9404 (IntE_2-tefInt->AtABCG25_Prtefint->Nat) | This study |
| ST9727 | ST9726 DTX50<-GPD-tefInt->BcABA3_Prtefint->Nat | ST9726 | pCfB9401 (IntE_2-DTX50<-GPD-tefInt->BcABA3_Prtefint->Nat) | This study |
| ST9728 | ST9726 AtABCG25<-GPD-tefInt->BcABA3_Prtefint->Nat | ST9726 | pCfB9402 (IntE_2-AtABCG25<-GPD-tefInt->BcABA3_Prtefint->Nat) | This study |

**Supplementary table 2.** Plasmids used in this study.

| Plasmid Name | Parent plasmid | Biobricks | References |
| --- | --- | --- | --- |
| pBP8009 (pIntF_3-TPex20-TLip2) |  |  | (Holkenbrink *et al.* 2018) |
| pCfB3405 (pORI1001-Nat-CEN1-USER) |  |  | (Holkenbrink *et al.* 2018) |
| pCfB5119 (pIntB-HphMx-YlHMG1<-PrGPD-PrFBA1->YlGGS1) | |  | (Kildegaard *et al.* 2017) |
| pCfB5935 (pIntA-1-HphMx-TPex20-TLip2) |  |  | (Holkenbrink *et al.* 2018) |
| pCfB6605 (pIntE-4-Hph-PrExp->YlSQS1) |  |  | Unpublished |
| pCfB6620 (pORI1001-Nat-CEN1-USER-IDI1<-PrEXP-PrGPD->ERG20) | |  | Unpublished |
| pCfB6633 (pNat-YLgRNA2_IntE_1) |  |  | (Holkenbrink *et al.* 2018) |
| pCfB6638 (pNat-YLgRNA2_IntE_4) |  |  | (Holkenbrink *et al.* 2018) |
| pCfB6677 (pIntE_1-TPex20-TLip2) |  |  | (Holkenbrink *et al.* 2018) |
| pCfB6679 (pIntE_4-TPex20-TLip2) |  |  | (Holkenbrink *et al.* 2018) |
| pCfB6681 (pIntE_3-TPex20-TLip2) |  |  | (Holkenbrink *et al.* 2018) |
| pCfB6681 (pIntE_3-TPex20-TLip2) |  |  | (Holkenbrink *et al.* 2018) |
| pCfB7063 (prDNA-Ura3d1-TPex20+PTEfintron+CrtW+TLip2) | |  | (Kildegaard *et al.* 2017) |
| pCFB8843 (pORI1001-Hyg-CEN1-USER) |  | BB3924 (Episomal vector backbone w/o HphMX):BB3925 (HphMX-TTef1 insert) | (Arnesen *et al.* 2020) |
| pCfB8878 (IntD_1-IDI1<-PrGPD-PrTefInt->ERG20) |  |  | (Arnesen *et al.* 2020) |
| Integration plasmids | | | |
| pCfB8870 (IntE_3-PrTefInt->POS5_YAli0e17963G) | pCfB6681 (pIntE_3-TPex20-TLip2) | BB3879 (Tefint->):BB3945 (POS5_YAli0e17963G) | This study |
| pCfB8946 (IntE_4-BcCPR1<-PrGPD-TefInt->BcABA3) | pCfB6679 (pIntE_4-TPex20-TLip2) | BB3865 (<-PrGDP_Tefint->):BB4002 (BcCPR1):BB4004 (BcABA3) | This study |
| pCfB8947 (IntE_4-BcCPR1<-PrGPD-TefInt->BcABA5) | pCfB6679 (pIntE_4-TPex20-TLip2) | BB3865 (<-PrGDP_Tefint->):BB4002 (BcCPR1):BB4003 (BcABA5) | This study |
| pCfB8948 (IntE_1-BcABA1<-PrGPD-TefInt->BcABA2 ) | pCfB6677 (pIntE_1-TPex20-TLip2) | BB3865 (<-PrGDP_Tefint->):BB3998 (BcABA1_GPD/TpexF):BB4000 (BcABA2) | This study |
| pCfB9108 (IntE_3-TefInt->BcABA4) | pCfB6681 (pIntE_3-TPex20-TLip2) | BB3879 (Tefint->):BB3997 (BcABA4) | This study |
| pCfB9109 (IntF_3-TefInt->BcABA4) | pBP8009 (pIntF_3-TPex20-TLip2) | BB3879 (Tefint->):BB3997 (BcABA4) | This study |
| pCfB9122 (IntE_3-TefInt->HMG) | pCfB6681 (pIntE_3-TPex20-TLip2) | BB3879 (Tefint->):BB4100 (HMG) | This study |
| pCfB9123 (IntE_3-TefInt->BcABA5) | pCfB6681 (pIntE_3-TPex20-TLip2) | BB3879 (Tefint->):BB4003 (BcABA5) | This study |
| pCfB9124 (IntE_3-TefInt->BcABA1) | pCfB6681 (pIntE_3-TPex20-TLip2) | BB3879 (Tefint->):BB3999 (BcABA1_Tefint/TlipF) | This study |
| pCfB9125 (IntE_3-TefInt->BcABA2) | pCfB6681 (pIntE_3-TPex20-TLip2) | BB3879 (Tefint->):BB4000 (BcABA2) | This study |
| pCfB9126 (IntE_3-TefInt->BcCPR1) | pCfB6681 (pIntE_3-TPex20-TLip2) | BB3879 (Tefint->):BB4101 (BcCPR1) | This study |
| pCfB9127 (IntE_3-TefInt->Bcin01g03510) | pCfB6681 (pIntE_3-TPex20-TLip2) | BB3879 (Tefint->):BB4001 (Bcin01g03510) | This study |
| pCfB9128 (IntE_3-TefInt->ERG20) | pCfB6681 (pIntE_3-TPex20-TLip2) | BB3879 (Tefint->):BB3871 (ERG20->) | This study |
| pCfB9192 (IntE_3-TefInt->BcABA3) | pCfB6681 (pIntE_3-TPex20-TLip2) | BB3879 (Tefint->):BB4004 (BcABA3) | This study |
| pCfB9399 (IntE_3-ERG20<-GPD-tefInt->BcABA1) | pCfB6681 (pIntE_3-TPex20-TLip2) | BB3865 (<-PrGDP_Tefint->):BB3999 (BcABA1_Tefint/TlipF):BB4407 (ERG20_GPD) | This study |
| pCfB9400 (IntA_1-POS5<-GPD-tefInt->BcABA4_PrExp->Hph) | pCfB5935 (pIntA-1-HphMx-TPex20-TLip2) | BB3865 (<-PrGDP_Tefint->):BB3997 (BcABA4):BB4406 (POS5_GPD) | This study |
| pCfB9401 (IntE_2-DTX50<-GPD-tefInt->BcABA3_Prtefint->Nat) | pCfB4779 (pIntE_2-Nat-TPex20-TLip2) | BB3865 (<-PrGDP_Tefint->):BB4004 (BcABA3):BB4408 (DTX50_GPD) | This study |
| pCfB9402 (IntE_2-AtABCG25<-GPD-tefInt->BcABA3_Prtefint->Nat) | pCfB4779 (pIntE_2-Nat-TPex20-TLip2) | BB3865 (<-PrGDP_Tefint->):BB4004 (BcABA3):BB4409 (AtABCG25_GPD) | This study |
| pCfB9403 (IntE_2-tefInt->DTX50_Prtefint->Nat) | pCfB4779 (pIntE_2-Nat-TPex20-TLip2) | BB3879 (Tefint->):BB4410 (DTX50_tefint) | This study |
| pCfB9404 (IntE_2-tefInt->AtABCG25_Prtefint->Nat) | pCfB4779 (pIntE_2-Nat-TPex20-TLip2) | BB3879 (Tefint->):BB4411 (AtABCG25_tefint) | This study |
| pCfB9666 (pIntE_2-Nat-Tefint->BcABA1) | pCfB4779 (pIntE_2-Nat-TPex20-TLip2) | BB3879 (Tefint->):BB3999 (BcABA1_Tefint/TlipF) | This study |
| pCfB9679 (pIntE_2-Nat-Tefint->BcABA3) | pCfB4779 (pIntE_2-Nat-TPex20-TLip2) | BB3879 (Tefint->):BB4004 (BcABA3) | This study |
| gRNA-plasmids | | | |
| pBP8003 (pNat-YLgRNA4-IntF_3) |  |  | This study |
| pCfB6637 (pNat-YLgRNA3_IntE_3) |  |  | (Holkenbrink *et al.* 2018) |
| pCfB6638 (pNat-YLgRNA2_IntE_4): |  |  | (Holkenbrink *et al.* 2018) |
| pCfB8858 (pHphM-YLgRNA2_IntE_1) | pCFB8843 (pORI1001-Hyg-CEN1-USER) | BB3929 (gRNA-cassette for IntE_1) | This study |
| pCfB8861 (pHphM-YLgRNA2_IntE_4) | pCFB8843 (pORI1001-Hyg-CEN1-USER) | BB3932 (gRNA-cassette for IntE_4) | This study |
| Synthetic genes | | | |
| pCfB8914 (BcABA1) |  |  | This study |
| pCfB8915 (BcABA2) |  |  | This study |
| pCfB8916 (BcABA3) |  |  | This study |
| pCfB8917 (BcABA4) |  |  | This study |
| pCfB8918 (BcCPR1) |  |  | This study |
| pCfB8919 (BcABA5) |  |  | This study |
| pCfB8920 (Bcin01g03510) |  |  | This study |
| pCfB9397 (DTX50_YlOp) |  |  | This study |
| pCfB9398 (AtABCG25_YlOp) |  |  | This study |

**Supplementary table 3.** Biobricks used in this study.

| Biobrick Name | Template | Forward primer | Reverse primer | Reference |
| --- | --- | --- | --- | --- |
| BB1635 |  |  |  | (Holkenbrink *et al.* 2018) |
| BB1636 |  |  |  | (Holkenbrink *et al.* 2018) |
| BB3863 (Tefint(PrGDPfusion)->) | pCfB7063 | 24013 | 18214 | This study |
| BB3864 (<-PrGDP) | pCfB5119 | 15528 | 15529 | This study |
| BB3865 (<-PrGDP_Tefint->) | BB3863+BB3864 | 15528 | 18214 | This study |
| BB3871 (ERG20->) | pCfB6620 | 24022 | 24023 | This study |
| BB3879 (Tefint->) | pCfB7063 | 23847 | 18214 | This study |
| BB3924 (Episomal vector backbone w/o HphMX) | pCFB3405 | 23934 | 10593 | This study |
| BB3925 (HphMX-TTef1 insert) | pCfB6605 | 23935 | 23936 | This study |
| BB3929 (gRNA-cassette for IntE_1) | pCfB6633 | 10607 | 10604 | This study |
| BB3932 (gRNA-cassette for IntE_4) | pCfB6638 | 10607 | 10604 | This study |
| BB3945 (POS5_YAli0e17963G) | ST3683 gDNA | 24368 | 24369 | This study |
| BB3997 (BcABA4) | pCfB8917 | 24393 | 24394 | This study |
| BB3998 (BcABA1_GPD/TpexF) | pCfB8914 | 24395 | 24396 | This study |
| BB3999 (BcABA1_Tefint/TlipF) | pCfB8914 | 24397 | 24398 | This study |
| BB4000 (BcABA2) | pCfB8915 | 24399 | 24400 | This study |
| BB4001 (Bcin01g03510) | pCfB8920 | 24401 | 24402 | This study |
| BB4002 (BcCPR1) | pCfB8918 | 24403 | 24404 | This study |
| BB4003 (BcABA5) | pCfB8919 | 24405 | 24406 | This study |
| BB4004 (BcABA3) | pCfB8916 | 24407 | 24408 | This study |
| BB4100 (HMG) | pCfB5119 | 24916 | 24917 | This study |
| BB4101 (BcCPR1) | pCfB8947 | 24918 | 24919 | This study |
| BB4147 (BcABA2_GPD/Tpex) | pCfB8948 | 25062 | 25063 | This study |
| BB4406 (POS5_GPD) | pCfB8870 | 26443 | 26444 | This study |
| BB4407 (ERG20_GPD) | pCfB8878 | 26445 | 26446 | This study |
| BB4408 (DTX50_GPD) | pCfB9397 | 26451 | 26452 | This study |
| BB4409 (AtABCG25_GPD) | pCfB9398 | 26453 | 26454 | This study |
| BB4410 (DTX50_tefint) | pCfB9397 | 26455 | 26456 | This study |
| BB4411 (AtABCG25_tefint) | pCfB9398 | 26457 | 26458 | This study |

**Supplementary table 4.** Primers used in this study.

| Primer Name | Sequence (5'->3') |
| --- | --- |
| 10593 | AGCAGGCTUGGAGGCGACGTGGCAG |
| 10604 | ACGCGAUACCGTACCCACACAAAAAAAGCACCACCGACTC |
| 10607 | CGTGCGAUAGTGAATCATTGCTAACAGATC |
| 15528 | ATGACAGAUTGTTGATGTGTGTTTAATTCAAGAATG |
| 15529 | AGCTACTGAUGACGCAGTAGGATGTCCTGCACGG |
| 18214 | AGTACTGCAAAAAGUGCTG |
| 23847 | CGTGCGAUAGAGACCGGGTTGGCGGCGCAT |
| 23934 | ACCCATTGCTGUAGATATGTCTTGTGTGTAAGGGGG |
| 23935 | ACAGCAATGGGUAAAAAGCCTGAACTCACCGC |
| 23936 | AAGCCTGCUGAATTCGGACACGGGCAT |
| 24013 | ATCAGTAGCUAGAGACCGGGTTGGCGGCG |
| 24022 | ACTTTTTGCAGTACUAACCGCAGTCCAAGGCGAAATTCGAAAGC |
| 24023 | CACGCGAUCTACTTCTGTCGCTTGTAAATCTT |
| 24368 | ACTTTTTGCAGTACUAACCGCAGCGACTACTCATCCGCCGAA |
| 24369 | CACGCGAUCTAAGCAACATCGCCTGACG |
| 24393 | ACTTTTTGCAGTACUAACCGCAGTCCTCCCAGCCCTTCACC |
| 24394 | CACGCGAUCTAACATCGCCAACCACCGT |
| 24395 | ATCTGTCAUGCCACAATGTCCAACTCCATCCTGAAC |
| 24396 | CGTGCGAUCTACTTGTACTCTGTGCCCTC |
| 24397 | ACTTTTTGCAGTACUAACCGCAGTCCAACTCCATCCTGAACCTG |
| 24398 | CACGCGAUCTACTTGTACTCTGTGCCCTC |
| 24399 | ACTTTTTGCAGTACUAACCGCAGCTGCTGTCTATCAAGGACCTG |
| 24400 | CACGCGAUCTATCGGGGGACCTCCTTG |
| 24401 | ACTTTTTGCAGTACUAACCGCAGAACTCCTACTACACCTTCTGGT |
| 24402 | CACGCGAUCTATCGGACGACGGCCTG |
| 24403 | ATCTGTCAUGCCACAATGGGCGGCCAACTGGAC |
| 24404 | CGTGCGAUCTAGGACCACACGTCCTCC |
| 24405 | ACTTTTTGCAGTACUAACCGCAGGCCACCCTGGTGGAGACC |
| 24406 | CACGCGAUCTACTCGAATCGCTGGTATCGA |
| 24407 | ACTTTTTGCAGTACUAACCGCAGCAGCAGGTGATCACCCAGA |
| 24408 | CACGCGAUCTAGACAGGGACCTCGAAGT |
| 24916 | ACTTTTTGCAGTACUAACCGCAGCTACAAGCAGCTATTGGAAAGAT |
| 24917 | CACGCGAUCTATGACCGTATGCAAATATTCGA |
| 24918 | ACTTTTTGCAGTACUAACCGCAGGGCGGCCAACTGGACGTG |
| 24919 | CACGCGAUCTAGGACCACACGTCCTCCTGG |
| 25062 | ATCTGTCAUGCCACAATGCTGCTGTCTATCAAGGACCT |
| 25063 | CGTGCGAUCTATCGGGGGACCTCCTTGA |
| 26443 | ATCTGTCAUGCCACAATGCGACTACTCATCCGCCGA |
| 26444 | CGTGCGAUCTAAGCAACATCGCCTGACG |
| 26445 | ATCTGTCAUGCCACAATGTCCAAGGCGAAATTCGAAAG |
| 26446 | CGTGCGAUCTACTTCTGTCGCTTGTAAATCTTG |
| 26451 | ATCTGTCAUGCCACAATGTCCCAGTCTAACCGAGT |
| 26452 | CGTGCGAUCTACTTGTCGACCATGCCG |
| 26453 | ATCTGTCAUGCCACAATGTCTGCCTTCGACGGTG |
| 26454 | CGTGCGAUCTAGTGCTTAATTCGTCGAAGAG |
| 26455 | ACTTTTTGCAGTACUAACCGCAGTCCCAGTCTAACCGAGTCC |
| 26456 | CACGCGAUCTACTTGTCGACCATGCCG |
| 26457 | ACTTTTTGCAGTACUAACCGCAGTCTGCCTTCGACGGTGTGG |
| 26458 | CACGCGAUCTAGTGCTTAATTCGTCGAAGAGC |

## Synthetic genes

pCfB8916 (BcABA3)

ATGCAGCAGGTGATCACCCAGACCCTCGTGGATGACCGATTCATTCAAATCTCTGACTCCAAGAAGTCCGAGGGCCTTGCTACTGATTCTACCAAGCGACAGTCCCAGGAGCAGCCCATTCACGATAAGGACCCCATTAAGGCTGCCACCGCTGCCATGGCCGCTACGCCCCTGGTGAAGGAGCACCAGGACACATGGTACTACCCGCCCGACATCGCCAACGACCTTCAGTCCATCAACCTGCCTGCCGAGCTCAAGGGTGAGATCTTCGCCTGCGCCTGGGAGTACACCCGATGTGTGATTCCTAACTACACCAACTGGAACCGATACGTTGCTTTTATGCGAATCATCATCATGGGTATCATTGCCGAGTTCCGAGGTGAGATGGTGGACGTCACCGCCTCCAATAACCTCCTGGGCTACGACCTCGACGCCACACTGGCTGCCCTTTTTGAGGGCACCCCCGGTCACAAAGAGATGGCTCGGGAGTACAAGACCTTCCTCCTTATCACTGCTGATAAAGCTTCCGAGCGACGAGACGGCGAGCTCTTCCGACGATACGTCAACGCTCTCGCCCAGTCTCCCCGACACTGGTTTCGAATGCGAGACTGTGACGCCCTGGCTCGTTTTACCATCGCCTCTGCTCTGGCTTGTAACGACTTGGACGATATCTGGTTCACCGAGGACCAGTTCGAGATCCTTACTGAGATTGGCGACACCCTGTACGATGCTGTGGCCTTTTACAAGCACCGAGCCGAGGGTGAGACCAACTCGACCTTCGCCTACATGCCCGAAGATCTTCGAATTAAGGCTTACTCTGAGTGTCGAGAGATCCTGTGGGCCCTGGATGCTGCCTGGGCTCGAAACCCCAAGCTGGCCAACGTCATCAACTTCGTCCGATTCTTCGGTGGTCCCATCCACATGATGATGCGACGATACAGATTCGTTGAGGAGAACCTGACCATCGGCAAGTCCGAGACTGACAAGGTCGTTGACCAGACCCGAAAGAACTTCAAGCTCTGGAATAGAGTGGACGCTAACAAGCGATCTGTTCTTAACACCCAGCGATACAAGGCCCTGATCGCCCGATCTGAGGAACTGATGTTCCCCGGCCTGGCCGAGTTTCTTGAGATGGGAGGTGACGGCATCTGCGACAAGTGCAAGTACCGAGAGTCTTACGGCGCTGAGCTGTCCCACCAGTTTGGTGGTGTCGAGCTGTGCTCCGAGTGTCGACTGTCCTGGCGAAAATACCTGGAGTGCTTCGTCGAGCGCGCCACGAAGGTCTTCCCTGAGCTGAAGACCCACTTCGAGGTCCCTGTCTAG

pCfB8914 (BcABA1)

ATGTCCAACTCCATCCTGAACCTGGGCTCTTTCGCTTGCCTTCTTTCCCTCGGTTCCATTGTCCTGTGGTACACTATCTCTGCCGTTCTCGCCTGGTACCCCCTGCGAAAGATCCCCGCCCCCTCCTTCCTTGCCACCTTCTCTTACCTGTGGCTGGCCAAGACCACCTACTCCGGTAAGCAGTACTGGATCCAGCGAGACCTGCATAAGAAGTACGGACCCCTGGTCCGAATCGGACCTACCGATATCATCACCGACGACCCCGAGATCATCAAAAAGATCTCCTCCGCTCGATCCTCTCACCGGCGAGGTGACTGGTACCTGACCGGTCGATTCAACCCTTACTACGACAACATGTTTACCATGCTGGAGCCTGGCCCACACGCCAAGGCAAAGGCACGAACCGCCGCAGCCTACTCGGGCCGCGACATGCCCGACCTGGAGGTTGGCGTGAACGCCCAGCTTCAGACTCTTATCGGTCTGATGCGATCCAAGTACGCTTCTAACACCGTGAAGCCCCACCAGCCCCTTCTAGACCTGGGTCAGGTCTCCTGCTTCTTCACTATGGATGTTATTACCAGACTGGCCTTCGGTGAAGAGTTCGGATACCTGAAGGAAGAGACCGACCAGTACGGTTTCCTGGGTGAAGTGCGAGAGCTGTGGCCCCGAATGTCTACCTCTGCTGACACTCCTTGGATCCGAAAGTTCCTGTTCTCCCCTCCCTTTCTCAAGGTGCTGGGTCCCAAGCCCACCGATAAAACCGGTTTTGGCGCTCTGATGGCCGTCGCCGAGCACCACGTCGGCAAGCGATTTGCCCCCGACGCCAAGAAGAAGGAGGATATGCTCGGCTCCTTCATCCGACACGGACTTAACCAGCAGGAGTGCGAGGTCGAGGGCCTTTTTATGATCGTCGCTGGTACCGAGTCTACTGCCTCCGCCATCCGGTCTACCCTTGTCCACGTGATGACCTGTCCCCGAGTTTACCAGAAGCTCAAGACCGAGATTAACCTGGCTGTCGAAGAGGGAAAGGTCTCTTCCCCCATTAAGCTTGAAGAGGCTAAGCTGCTGCCTTTTCTCCAGGCCGTTATTTACGAGGGCATCCGAATGCGACCCCCACTGCTGGGACTGTTCCCTAAGATCGTGCCAGACGGTGGAGAGGAGTTCCATGGCATGTTCATTCCCGCCGGAACCGCCATTTGTATGAACACCTCCTCCCTGCTGCGATCTACTGCCCTGTTCGGCGACGATGCTGAGGTCTACAGACCCGAACGATTCATGGAGCTGGAGAAGTCCAAGCGTGGTGAGATGGAGCGAAACGTCGAGCTCGCTTTCGGCTACGGCCAGTACATGTGCGTCGGTAAGACCGTTGCTTTTATGGAGCTAAATAAGTCTATCTTCGAGATTCTGCGAGCTTTTGACCTGCAGCTCCTGTCTCCCGCCAAGCCTTGTGACGTTCTGTCCTACGGCATCTTCCTTGAATCCAACATGCTTGTGAAGGTGACCGAGTCTGAGGGCACAGAGTACAAGTAG

pCfB8915 (BcABA2)

ATGCTGCTGTCTATCAAGGACCTGTCCGAGAAATACATTATGCTACTGGACGTTAAGGACCTCTCTACCCTGAAGACGACCGTCGCCGTCCTGGTCACAGTCGCCCTGATTGCTCAGGTTCTGTGGAAGATCTTCTTCCACCCCCTGTCCGCCTTCCCCGGTCCTTGGTTCAACCGAATCTCCGAAATCCCCGGCTCCTGGGTCATCGCCACCGGTAAGCAGCACTCCTACTACCGAAAGCTGCACGAAAAGTACGGACCTGTTGTCCGAGTCGCCCCCAACGAGTTGTCCTTCATTGGCGACCGGGCTTGGGACGACATCTACGGCGTCCAGAAGAAGGGCCCTAACTTCGAGAAGTCGCCCATCTTCATCGGTGCTGTGTCTCCCCTCGACGGACAGACCGGCATTTCCCTTGCTCCTAACGAGGCCCACACCCGACAGCGACGGGCCCTGGCCCACGTCTTCTCTAACACCGCTCTGCTCCAGCAGGAGGAGATCATGCGATCTCACGTCGACAAGCTGGTTGGTCAGCTTAAGAAGACCATTGCCGAGAACCGACCCATCAACTTCTCTAACTGGTACACCTACACTACCTTTGACATGATGGGCGACCTCTGCTTTGCTGAGCCTTTCGGTTGCCTGGACCAGGGAGGCGCCACTGAGTGGTCCACCTCTGTCATTAACGTCTTCAAGTCTGCCGCTTGGGACCAATCCATCCGAAGGGTGGCCGGTGTGAATACTTGGCTTCAAAAGCTGATGGTCAAGCTGCTGATCCCGTCGAAGGCGGCTAACTGGCGAAAGGTCCATTTCCAAAATTCTCGGGAAAAGACCCTGCGACGACTCGCCGATGGAAACCGAGAGCATAAGGACTTCATCTACCACATCCTGAAAAACAAGGAAGCCAAGAACTCTCTGTCTGAGACTGAGATTATTCTTAACATGGTCCTGCTGATCTCCGCCGGTACCGAGACCACCGCTTCCCTGCTGACCGGTTGGACCTACTTTATCTGCACCCATCCCGAGGTGTATAAGCGACTGACCGATGAGATCCGAGGCCGATTCAACTCCGAGCAGGATATCACCTGGGAGACTGTCAAGGATCTTCCTTATCTGCATGCCACCCTCTCCGAGGCTCTCCGACTTTACTCGCCAGCCCCCGCCAACCAGCAGCGAATTGTCCCTCCCGGCGGTTCTGTCATCGACGGCCACTTCGTCCCCGGTAAAACCACTGTGGCCGTTGCCCCCTGGGCTGCCATTAACTCCTCCCTGAACTTCAAGGACCCCCAGAAATTCATCCCCGAGCGATGGCTCGGTGACGAGCGATTCGTCAACGACAAGCTTAACGCCTCTCAGCCTTTCTCCCTCGGCCCTCGAGGTTGCATCGGCAAGAACCTCTCCTTCTTCGAGATGCGACTCATCACCTCTCGACTCCTGTGGAACTTTGACGTCTCCCTTGTCACCACCGGCGAACACGGTGAGACCAACAAGCTCTGGGACATGGACGGAGCTGGTAAGTATATGAAGGTCTACCAGACCTGGAATAAGCCCGATATGTGGGTCATGCTCAAGGAGGTCCCCCGATAG

pCfB8917 (BcABA4)

ATGTCCTCCCAGCCCTTCACCAACAAGGTGATTGCCCTGACCGGTAGCGCCTCCGGCATCGGCCTTGAGACCGCCAAGCTGCTTGCCTCCCGGGGCGCCCGACTGTCTCTGGCCGACATCCAGGAGGACAAGCTTAAGGAGCTCCAGGCCCAACTTGAGTCTGAATATTACGTCGATGTCATCACCACTAAGGTCGATGTCCGAAAGTTCGGTGAGGTTGAGGCCTGGATCAACAAAACTATTGACAACTTCGGCAAGCTGGACGGATCTGCCAACCTGGCTGGCGTCGCCCCTGAGTCTATCGGACTGAAGGGCATCGTCGAGCAGGACCTCGACGAGTGGGAGTTTGTCCTGGGCGTCAACCTCACCGGTACCATGAACTCCCTGAAGGCCCAGCTGAAGGTCATGGCCAACAACGGTTCTATCGTCAACGCCTCCTCTATTCGAGGCCTTACCGGTGCAGCTAAGAACGCTTCCTATTCCTCTGCTAAGCACGGCATCATCGGTCTGACCCGAACTGCTGCCAAGGAGGTCGGCGGCAAGGGCATCCGCGTGAACGCCATTTGTCCCGGCCGAATCTCCACCCCCATGCTGAAGACCGCTGAGAACTCCATTGGCCTGCACCTGCAGCCCGGGTCTGCTAACTACCCTCCCATCGCCCTGGGTCGAGACGGCGAGGCTAAGGAGGTGGCTCAGCTTGTCGCCTTTCTGCTGTCTGACGAGTCCACCTACATCTCCGGCGCAGACATCTCCATTGACGGTGGTTGGCGATGTTAG

pCfB8918 (BcCPR1)

ATGGGCGGCCAACTGGACGTGCTCGACATCGTTGTCCTGGGTGTCTTAGCTCTTGGTACCATCGCTTACTTTACCAAGGGCAAGTATTGGGGTGTCGTTAAGGACCCCTACGCTACTTCTTACGCCGCTACTAACGGCAACAAGCCTGCTAAGACTCGAAATATCACTGAGAAGATGGACGAGTCGAACAAGAACTGCGTGGTGTTCTACGGCTCTCAAACTGGCACCGCTGAGGATTACGCCAGCCGACTCGCCAAGGAGGGTAAGTCTCGGTTCGGTCTGGAAACTATGGTGGCCGACCTGGAAGATTACGACTATGACACCCTGGACACCTTCGGCGAAGATAAGGTCGCTATTTTCGTCCTCGCCACTTACGGCGAGGGTGAACCCACTGACAACGCTGTTGACTTTTACGAATACTTCATGAACGAGGATGTGGAGTTCTCCTCGGGTGAGAAGTCTCTTGCCAACCTCAAGTTCGTCGCTTTCGGCCTGGGCAACAACACCTATGAGCACTACAACTCCATGGTCAGAAACGTCACCAAGGCTTTTGAGAAGCTCGGAGCTACCAGAATTGGTGAAGCTGGTGAGGGCGACGACGGCGCTGGAACCATGGAGGAGGATTTCCTCGCTTGGAAGGACCCTATGTGGACCGCCCTCGCTGAAAAGATGGGCTTACAGGAGCGAGAAGCCGTGTTCGAGCCCGTGTTTGCCATTACCGACCGAGAGGAGCTTACCAAGGACTCCTCGGAGGTCTACCTGGGTGAGCCTAATAAAATGCATTTAGAGGGAACCTCCAAGGGCCCTTACAACGCCCATAACCCCTACATTGCCCCCATTTCTGAGTCCAAGGAGCTCTTCACCGTCAAGGATCGAAACTGCTTGCACCTAGAGATCGACATTTCCGGCTCCAACCTCTCCTACCAAACTGGAGACCACATCGCTGTCTGGCCCACGAACGCCGGCAGGGAGGTCGATCGATTCCTGGACGTCACTGGACTCTCTTCCAAGAAGGACAGTGTCATTACCGTCAAAGCTCTTGACTCCACGGCCAAGGTCCCCTTCCCAACTCCCACCACCTACGACGCCATCGTCCGATATCATATGGAAATCTGTGCTCCTGTTTCCAGACAGTTCCTGGCCACCCTGGCCGCATTCGCCCCCAATGACGCCATTAAGGCCGAGATGGAGAAACTCGGTGGCGACAAAGACTACTTCCACCAGAAGATTTCGAACAACTACCTGAACATCGCCCAAGTGCTGCAGGAGGTCGGTGGTCAGGAAAAGTGGTCCGCTATTCCTTTTTCCGCCTTCATTGAGGGTTTGGGCAAGATCCAGCCCCGATACTACTCCATCTCCTCCTCGTCCCTGGTGCAGAAGAAGAAGATCTCTATCACAGCCGTGGTGGAGTCTACTGACATCCCCGGCCGAACTGACGCCCTGAAGGGCGTTACCACCAACTACCTCCTGGCCCTCAAGCAGAAGCAGCATGGTGACGAGCACCCAGACCCCCATGGATTGACCTACGAGATCACCGGTCCCCGGAACAAGTACGACGGTATCCACGTGCCTGTGCACGTGCGACATTCCAACTTCAAGCTGCCCTCTGACCCTTCTAAGCCTGTGATCATGATCGGACCTGGTACCGGTGTCGCCCCTTTCCGAGCTTTTGTCCAAGAGCGAGCTGCCCAGGCCAAGGCCGGAGAGAACGTCGGTCGAACTATTCTGTTCTTCGGCTGTCGAAAGTCCACCGAGGACTTCATGTATAAGGATGAGTGGAAGGAGTATGAGGAGGCTCTTGGCGACAAGTTCTCTCTGATCACCGCCTTCTCTCGAGAAGGTAAGGAGAAGGTCTACGTGCAGCACCGACTGAAGGAGCACGCAAAGGAGATCAACGACCTGCTTATGCAAAAGTCTTACTTCTACGTGTGCGGAGACGCTGCCAACATGGCTCGAGAGGTCAACACCGTCCTCGGCCAGATCATTAGTGAGCAGCGAGGAATTCCCGAGTCGAAGGCAGAGGACATCGTTAAGTCCATGCGGAGCGCCAACCAGTACCAGGAGGACGTGTGGTCCTAG

pCfB8919 (BcABA5)

ATGGCCACCCTGGTGGAGACCCAGATCTCCTCCGTCTACCACTCTATTGACACCTCTAAGACTCACCCCCACTTCTCTCGATTCCCTGCCGCCATTTCCATCGCCAACGATGAGATCGAACAGACCTTGCGAGAGCTGGGCGAGCGGGCTACCGAGCCCGGTACCCGAGTCAGACGACGAATTCAGATCAGACACACTTGCCCCTACGGCGACCCTTTCGGCATCTGTCATGCTTCCGCCTTCCCCGAGCGACTTGTCCTTCTGGGTTCCATCGTCGAGATCATGTGGGTTCACGATGACATCACCGAGGAGATTGACCTGAAGGAGGCTATGGAGCAGCATGAGCTGCTGAAGAAGGTGCTGACTACTGACATTGATTCTAAGACCTTCGAGTTCCAGAACGATCGACAAGTTCTGCTGGCCAACATTCTGCAGAAGGCCATCCAGATGGACCCCGAGGCCGCCCCCACAATGATCCAGACCCTCCGAAACTACCTTGACACCTTTGATAACCGAGACGATGACTTCGACACCATGTCCGAGTACATGCCCTACCGAATCGCCAACTGCGGTTACTGGATCTCCTCTTACTTCATCCGATGGGGTATGGGAACCATTCTCTCCGAGGAAGACTACAAGTCCATCCGAGAGTACGACATCACCATGGGTAACATCCTCGGTCTTACCAACGACTACTTCTCCTGGCACGTCGAGAAGGACCAGCCCACCGGCCGAATCCGAAACGGCGTCCGAGTCCTGATGAAGCAGCACAACATCCCCGCCGAGATCGCTCAGAAGCTTCTGCTGGGCATCATTATCGAGGAGGAGTCCAAAGCCGTTAAGCTGCGAGAGGAGCGACTCAAGACCCACGCCTCCCAGTCTGTCCTGGAGTACATCAAGGCCATTGAGCTTTATGTCGGCGGTTCTTGCTACTGGCACTCCACTGCCCCTCGATACCAGCGATTCGAGTAG

pCfB8920 (Bcin01g03510)

ATGAACTCCTACTACACCTTCTGGTACTCTCCTCAGGGTTCTAAGCAAGAGGCCGACCACTGGGCCTGGAACCTGGATTGGGCTTCTAAGCTGCTTCAGATCCTGGGCTTCCTGGTTGCCCTCGCTCTGTCTTGGTTCGCCGTCTGTCTCTACAAGGCCCGAAAGAAGTTCATCGTCCTTCGATCTAAGGGTATTCCCATTCCCCGATACTCTTACATTTTCGGACACCTGCCTGTGGTCCTCGACTTCCACCGAGAGTGGGCTATGGATGCCAACCTGACCCAGACTCTCGGTCTGTACATTGCTAAGAATTGGCAGAAGTTCTTCCCCGGTGAGCACCACCGCCCTCCCATTATCTACCTGGACCTCTGGCCCATCGCCGAGCCCCTCGCTATTTCCATCGACGTCTCTACCTCTAACCAGATGGTGTTCGACAAGATGAACCTTCCCAAGTCTCACATGGAGGGCGACTTCCTCATCCCCCTCACTAAGGGCAAGGACTTCGGTTCTCTCTCTGGCGATGAGTGGCGCTTCTGGCGATCCACCTTTAACCCCACCTTTGGAGCTGCTAATGTGGCTGCCCTGGTTCCCGCCATCTGCGAGGAAGTCGAGATGTTCACCCGAGCACTGCAGGACCGATGCGGCGATGGTGATACCTGGGGTCAGGTCTTCCCCTTTGAGAATGTGTCGGCCGAGCTGATCTTCGACATTACAGGCCGAGTCGTGATCGGTAAGCGACTGAACACCCAGTCTGCTAACCCAGAGCTGTTCTCTTCCCTGTTCCGAGCTCAGCTGACCCGAATGGAGATCACCCTGAACCCTGCTAAGGTTCTTTGGAGACTGACCCCCATGTCTAAGCGATACCTGACTAAGAAGCGAGACGAGATGCTGGAGTGGCTGCGACCTTTCATTCTGGAGTCTTTCCAGCGAGCTTCTTCCTCTGAGTCCGAGACCATCGTTGAGGCCGCTGCCAAGGAGCATAGAGAGAAGGTGTGGATCGACAAGGTGCAGGCTTCGTGTGACGAGTCCTTCATCGACGCCGTCTTCTGTCAGCTGATGATTTGCTTCTTTGCCTCTGACGATGCTCCCACCAGTACCATTCCCTGGCTGTTCGAGTACCTGCATCGAAACCCAGAGAGCCTCATCAAGCTGCGAGCTGAACACGACTCTGTCCTGGGTACCAACCCCGAAGCTGCCGCCAAAACTATCCGGGAGGAGCCCGAGCTCCTGAACCAGCTGCCTTACACCACAGCCGTGATCAAGGAGACCATCCGACTGTCCCCTGCCACCACCACAATCCGAGAGGGCCATCAGAACTTCAACTTCCACATCACTGGATTCGACAACAAGTGGCCTGAGTACTGGCCTTCTGCCGGTTTCGACCTTCTGGACTCTCCCCGAACCATTCACACCGACCCTAACATCTTTCTGAAGCCCCACGAGTTCATTCCCGAGCGATATCTGGTCGCTGATACAGACCCTCTCTACCCTCCTCCCAACGCCTGGCGAGGCTTCCAACTCGGACAGCGACGATGCATTGGCCAAGTCCTGGCCTACGCTGAGCTCAAGCTCGTCCTTGTCCTCACCGCTAGACGATTCGACATCGAACCTGCTTGGGAGGACTGGGATCTGATGCTCTCCCAGCAGGGCAAAAAGGTCAAGCCTCACCTTGTCGAGGGAGAGCGACTATACATCACCGGAAACGCCACCTCTCACTCTAAGGATGGTGCCCCCGTCCATGTCAGAGCCCGTAAGTTCAGCGCCTCCGAGGAGTCTCTGTTCGAAAACCAGGCCGTCGTCCGATAG

pCfB9397 (DTX50_YlOp)

ATGTCCCAGTCTAACCGAGTCCGAGATGAGGTGACCCTCCCTCTGCTGCAGAAGACTTCCCACCTGAAGAACCACTCTTCGGTTCTGTCCGTGTTCCTGAACGAGGCTATTTCCATTTGCAAGATTTCCTACCCCCTCGTTCTGACCGGCCTTTTCCTGTACGTGCGATCTTTCGTGTCCCTGTCCTTCCTGGGCGGTCTGGGTGATGCCACTCTGGCTGGCGGCTCGCTTGCCGCTGCCTTCGCTAACATCACCGGCTACTCCCTTTTCTCTGGTCTTACCATGGGTGTCGAGTCCATCTGTTCGCAGGCCTTCGGCGCCCGTCGATACAACTACGTTTGTGCTTCTGTTAAGCGAGGCATCATCCTGCTGCTCGTGACTTCCCTGCCCGTCACCCTGCTGTGGATGAACATGGAGAAGATCCTCCTCATCCTGAAGCAGGACAAGAAGCTGGCCTCTGAGGCCCACATCTTCCTCCTGTACAGCGTACCCGATCTGGTTGCTCAATCTTTCCTGCACCCCCTGCGAGTCTACCTGCGAACCCAGTCCAAGACCCTGCCTTTGTCCATTTGTACCGTTATCGCCTCCTTCCTTCACTTGCCCATCACTTTCTTCCTGGTGTCTTACCTGGGACTGGGCATCAAGGGCATTGCCCTGTCTGGAGTCGTCTCTAACTTCAACCTGGTCGCCTTCCTTTTTCTGTACATCTGCTTCTTTGAGGACAAGCTGTCCGTCAACGAAGACGAGAAGATTACCGAGGAGACCTGTGAAGACTCTGTGCGAGAATGGAAGAAGCTGCTGTGTCTCGCCATCCCTTCTTGCATCTCCGTCTGCCTCGAGTGGTGGTGCTACGAGATCATGATCCTCCTGTGTGGTTTCCTTCTTGACCCTAAGGCTTCCGTGGCCTCCATGGGTATTCTGATTCAGATTACTTCCCTGGTGTACATTTTCCCCCACTCGCTGTCCCTGGGTGTGTCCACCCGAGTCGGCAACGAGCTGGGCTCCAACCAGCCCAAGCGAGCCCGACGAGCCGCCATTGTTGGTCTGGGCCTGTCTATCGCTCTCGGCTTCACCGCTTTTGCTTTCACCGTCTCTGTCCGAAATACCTGGGCTATGTTCTTCACCGACGACAAGGAGATTATGAAGCTGACCGCAATGGCTCTGCCCATCGTGGGACTGTGTGAGCTGGGAAACTGCCCTCAGACCACCGGATGTGGCGTCCTTCGGGGTTCGGCCCGACCTAAGATTGGCGCCAACATTAACGGCGTGGCTTTCTACGCTGTGGGTATCCCTGTGGGCGCTGTCCTGGCCTTCTGGTTCGGTTTCGGCTTCAAGGGTCTGTGGCTGGGAATGCTGGCCGCCCAGATCACCTGCGTGATTGGTATGATGGCTGCTACCTGCCGAACTGATTGGGAGCTCGAGGCTGAGCGTGCTAAAGTGCTGACTACTGCTGTCGACTGCGGATCCTCTGACGACGATGCCAAGGAGGATATGGAGGCCGGCATGGTCGACAAGTAG

pCfB9398 (AtABCG25_YlOp)

ATGTCTGCCTTCGACGGTGTGGAGAACCAGATGAACGGTCCTGACTCCTCACCACGACTCTCTCAGGACCCCCGAGAGCCTCGATCTCTCCTGTCCAGCTCCTGCTTCCCCATCACTCTGAAGTTCGTCGACGTCTGCTACCGAGTTAAGATCCATGGTATGTCGAACGACTCTTGTAACATCAAGAAATTACTTGGACTCAAGCAGAAGCCCTCTGACGAGACCCGATCGACCGAGGAGCGAACCATTCTGTCCGGCGTTACCGGAATGATCTCCCCCGGTGAGTTCATGGCCGTGTTGGGTCCCTCTGGTTCCGGCAAGTCCACACTGCTGAACGCCGTGGCCGGTCGACTGCACGGCTCTAACCTTACCGGCAAGATCCTGATTAACGACGGAAAGATCACCAAGCAGACATTGAAGCGAACCGGCTTCGTCGCTCAGGACGACCTATTGTACCCTCACCTGACTGTCCGTGAGACTCTGGTCTTCGTTGCTCTGCTTCGACTCCCCCGATCGCTCACGCGAGACGTCAAGTTGCGAGCCGCCGAGTCTGTCATCTCTGAGCTGGGCTTGACTAAGTGCGAAAACACCGTCGTGGGCAACACCTTTATCCGAGGTATCAGCGGAGGTGAGCGAAAGCGCGTTTCTATCGCCCACGAGCTGCTCATTAACCCTTCCCTGTTGGTCCTGGATGAACCCACCTCCGGTCTGGACGCTACCGCCGCTCTCCGCCTGGTGCAGACTCTTGCTGGTCTGGCTCACGGTAAGGGCAAGACTGTCGTGACCTCCATTCACCAGCCCTCCTCCCGAGTCTTCCAGATGTTTGACACCGTACTCCTGCTGTCCGAGGGCAAGTGCCTCTTCGTCGGCAAAGGCCGAGACGCCATGGCTTACTTCGAGTCCGTCGGTTTTTCCCCCGCCTTCCCCATGAACCCCGCTGACTTTCTGCTCGACCTGGCCAACGGTGTTTGTCAGACCGACGGCGTCACTGAGCGAGAGAAGCCCAACGTGCGACAGACCCTCGTCACCGCCTACGACACCCTGCTCGCACCTCAGGTTAAGACCTGCATCGAGGTCTCTCATTTCCCCCAGGACAACGCTAGATTTGTGAAGACCCGAGTCAATGGTGGAGGTATCACTACCTGTATCGCCACCTGGTTCTCACAACTGTGTATCCTGCTCCACCGACTCCTGAAGGAGCGCAGACATGAGTCTTTCGACCTTCTGCGAATCTTCCAGGTGGTGGCTGCCTCTATCCTGTGCGGTCTGATGTGGTGGCACTCCGACTACCGAGACGTTCACGACCGACTGGGTTTGCTGTTCTTCATCTCCATCTTTTGGGGCGTGTTGCCCTCCTTTAACGCCGTTTTCACCTTCCCTCAGGAGAGAGCCATCTTCACCCGAGAGAGGGCTTCTGGCATGTACACTCTGTCCTCTTACTTCATGGCCCATGTTCTCGGCTCCCTGTCCATGGAGCTGGTGCTGCCAGCTTCTTTCTTGACCTTTACTTACTGGATGGTGTACCTGAGACCCGGAATCGTGCCTTTTCTGCTTACCCTGTCTGTCCTTCTGCTTTACGTTCTGGCCTCCCAGGGTCTGGGACTTGCCCTCGGTGCCGCTATCATGGACGCCAAGAAGGCCTCCACAATCGTCACGGTCACCATGCTTGCTTTCGTCCTGACGGGTGGCTACTACGTTAACAAGGTGCCCTCTGGCATGGTCTGGATGAAGTACGTCTCTACCACCTTCTACTGTTACCGACTTCTGGTCGCGATCCAGTACGGTTCAGGTGAGGAGATTCTTCGAATGCTCGGTTGCGACTCCAAGGGTAAGCAGGGCGCCTCTGCCGCAACTTCCGCTGGCTGTCGATTTGTGGAGGAGGAGGTCATCGGTGACGTCGGAATGTGGACTTCTGTTGGTGTCCTGTTTCTCATGTTCTTCGGCTACAGAGTGCTCGCCTACCTTGCTCTTCGACGAATTAAGCACTAG

**References:**

Angerer H, Radermacher M, Mankowska M *et al.* The LYR protein subunit NB4M/NDUFA6 of mitochondrial complex I anchors an acyl carrier protein and is essential for catalytic activity. *Proc Natl Acad Sci* 2014;**111**:5207–12.

Arnesen JA, Kildegaard KR, Cernuda Pastor M *et al.* *Yarrowia lipolytica* Strains Engineered for the Production of Terpenoids. *Front Bioeng Biotechnol* 2020;**8**:1–14.

Holkenbrink C, Dam MI, Kildegaard KR *et al.* EasyCloneYALI: CRISPR/Cas9-Based Synthetic Toolbox for Engineering of the Yeast *Yarrowia* *lipolytica*. *Biotechnol J* 2018;**13**:1-8.

Kildegaard KR, Adiego-Pérez B, Doménech Belda D *et al.* Engineering of *Yarrowia lipolytica* for production of astaxanthin. *Synth Syst Biotechnol* 2017;**2**:287–94.

Marella ER, Dahlin J, Dam MI *et al.* A single-host fermentation process for the production of flavor lactones from non-hydroxylated fatty acids. *Metab Eng* 2020;**61**:427–36.
